# Supplementary material for: Comparison of quality and interpretation of newborn ultrasound screening examinations for developmental dysplasia of the hip by basically trained nurses and junior physicians with no previous ultrasound experience
Source: PLoS One. 2024 Apr 18;19(4):e0300753. doi: 10.1371/journal.pone.0300753 (PMC11025947; doi:10.1371/journal.pone.0300753)
Supplement: Attachment — Submitted filename: PONE-D-23-02980-AF.pdf [file pone.0300753.s002.pdf]

PLOS ONE

Comparison of quality and interpretation of hip ultrasound screening examinations by basically trained nurses [illegible] junior physicians with no previous ultrasound experience

--Manuscript Draft--

Manuscript Number:	PONE-D-23-02980
Article Type:	Research Article
Full Title:	Comparison of quality and interpretation of hip ultrasound screening examinations by basically trained nurses to junior physicians with no previous ultrasound experience
Short Title:	Comparison of quality of hip ultrasound screening examinations by nurses to junior physicians
Corresponding Author:	Munkhtulga Ulziibat, M.D<br>National Center for Maternal and Child Health of Mongolia<br>Ulaanbaatar, MONGOLIA
Keywords:	hip ultrasound, Graf method, newborn screening, nurse study
Abstract:	Abstract<br>Background: Because resources are limited, adequately trained physicians are not always available, especially in remote rural areas. We are obliged to give babies in very rural areas of Mongolia the chance to profit from nationwide screening of developmental dysplasia of the hip. Thus, this study aimed to compare the quality and accuracy of newborn hip ultrasound screening examinations by nurses to junior physicians with no previous experience after standard training.<br>Methods: A group of 6 nurses and 6 junior physician volunteers with no previous ultrasound experience underwent Graf's standard training in hands-on practice and didactic instruction with prospective follow-up in urban and rural hospitals. Newborns were examined before discharge from the hospital, according to the national guideline. Two standard documentation images of each hip were saved digitally. The groups were compared on the proportion of good quality of sonograms and correct interpretation. Two Swiss supervisors' agreed diagnosis according to Graf was considered the final reference for the study purposes.<br>Results: A total of 201 newborns were examined in the study, with a mean age of 1.3±0.8 days at examination. Junior physicians examined 100 newborns, while nurses examined 101 newborns. The study subjects of the two groups were well balanced for the distribution of baseline characteristics, such as mean age, sex, birth weight, and hospitals.<br>The study observed no statistically significant difference in the quality of Graf's standard plane images between the providers: junior physicians and nurses (92.0% and 89.1%; p=0.484). Eventually, 92.0% (92) of the physician group and 89.1% (90) of the nurse group were correctly diagnosed as "Group A" (Graf's Type 1 hip) or "Non-Group A" hips (p=0.484). The most common errors among the groups were a missing lower limb, wrong measurement lines, and technical problems.<br>Conclusion: Our study provides evidence that after receiving standard training in Graf's hip ultrasound method, nurses can obtain standard hip sonograms according to Graf with equal quality as junior physicians. However, after basic training, regular quality control and refresher training are needed for nurses and physicians.
Order of Authors:	Munkhtulga Ulziibat, M.D
	Bayalag Munkhuu, PhD
	Raoul Schmid
	Corinne Wyder
	Thomas Baumann
	Stefan Essig, PhD
Opposed Reviewers:	
Additional Information:	

Powered by Editorial Manager® and ProduXion Manager® from Aries Systems Corporation

Question	Response
**Financial Disclosure**<br><br>Enter a financial disclosure statement that describes the sources of funding for the work included in this submission. Review the submission guidelines for detailed requirements. View published research articles from *PLOS ONE* for specific examples.<br><br>This statement is required for submission and **will appear in the published article** if the submission is accepted. Please make sure it is accurate.<br><br>**Unfunded studies**<br>Enter: *The author(s) received no specific funding for this work.*<br><br>**Funded studies**<br>Enter a statement with the following details:<br>• Initials of the authors who received each award<br>• Grant numbers awarded to each author<br>• The full name of each funder<br>• URL of each funder website<br>• Did the sponsors or funders play any role in the study design, data collection and analysis, decision to publish, or preparation of the manuscript?<br>• **NO** - Include this sentence at the end of your statement: *The funders had no role in study design, data collection and analysis, decision to publish, or preparation of the manuscript.*<br>• **YES** - Specify the role(s) played.<br><br>* typeset	Funding came from the supporters of Swiss Mongolian Pediatric Project (SMOPP) http://www.smop.ch/ and Swiss Association of pediatric Ultrasound (SVUPP) http:///www.svupp.ch/ the funders had no role in study design, data collection and analysis, decision to publish, or preparation of the manuscript.
**Competing Interests**<br><br>Use the instructions below to enter a competing interest statement for this submission. On behalf of all authors, disclose any competing interests that could be perceived to bias this work—acknowledging all financial support and any other relevant financial or non-financial competing interests.	The authors have declared that no competing interests exist.

Powered by Editorial Manager® and ProduXion Manager® from Aries Systems Corporation

This statement is required for submission and **will appear in the published article** if the submission is accepted. Please make sure it is accurate and that any funding sources listed in your Funding Information later in the submission form are also declared in your Financial Disclosure statement.<br><br>View published research articles from *PLOS ONE* for specific examples.<br><br>**NO authors have competing interests**<br><br>Enter: *The authors have declared that no competing interests exist.*<br><br>**Authors with competing interests**<br><br>Enter competing interest details beginning with this statement:<br><br>*I have read the journal's policy and the authors of this manuscript have the following competing interests: [insert competing interests here]*<br><br>* typeset	
**Ethics Statement**<br><br>Enter an ethics statement for this submission. This statement is required if the study involved:<br><br>• Human participants<br>• Human specimens or tissue<br>• Vertebrate animals or cephalopods<br>• Vertebrate embryos or tissues<br>• Field research<br><br>Write "N/A" if the submission does not require an ethics statement.<br><br>General guidance is provided below. Consult the submission guidelines for detailed instructions. **Make sure that all information entered here is included in the Methods section of the manuscript.**	Mongolian ethical committee, commissioned by the Ministry of Health

**Format for specific study types**<br><br>**Human Subject Research (involving human participants and/or tissue)**<br>• Give the name of the institutional review board or ethics committee that approved the study<br>• Include the approval number and/or a statement indicating approval of this research<br>• Indicate the form of consent obtained (written/oral) or the reason that consent was not obtained (e.g. the data were analyzed anonymously)<br><br>**Animal Research (involving vertebrate animals, embryos or tissues)**<br>• Provide the name of the Institutional Animal Care and Use Committee (IACUC) or other relevant ethics board that reviewed the study protocol, and indicate whether they approved this research or granted a formal waiver of ethical approval<br>• Include an approval number if one was obtained<br>• If the study involved *non-human primates*, add *additional details* about animal welfare and steps taken to ameliorate suffering<br>• If anesthesia, euthanasia, or any kind of animal sacrifice is part of the study, include briefly which substances and/or methods were applied<br><br>**Field Research**<br><br>Include the following details if this study involves the collection of plant, animal, or other materials from a natural setting:<br>• Field permit number<br>• Name of the institution or relevant body that granted permission	
**Data Availability**<br><br>Authors are required to make all data underlying the findings described fully available, without restriction, and from the time of publication. PLOS allows rare exceptions to address legal and ethical concerns. See the PLOS Data Policy and FAQ for detailed information.	Yes - all data are fully available without restriction

A Data Availability Statement describing where the data can be found is required at submission. Your answers to this question constitute the Data Availability Statement and **will be published in the article**, if accepted.<br><br>**Important:** Stating 'data available on request from the author' is not sufficient. If your data are only available upon request, select 'No' for the first question and explain your exceptional situation in the text box.<br><br>Do the authors confirm that all data underlying the findings described in their manuscript are fully available without restriction?	
**Describe where the data may be found in full sentences. If you are copying our sample text, replace any instances of XXX with the appropriate details.**<br><br>• If the data are **held or will be held in a public repository**, include URLs, accession numbers or DOIs. If this information will only be available after acceptance, indicate this by ticking the box below. For example: *All XXX files are available from the XXX database (accession number(s) XXX, XXX.*).<br>• If the data are all contained **within the manuscript and/or Supporting Information files**, enter the following: *All relevant data are within the manuscript and its Supporting Information files.*<br>• If neither of these applies but you are able to provide **details of access elsewhere**, with or without limitations, please do so. For example:<br><br>*Data cannot be shared publicly because of [XXX]. Data are available from the XXX Institutional Data Access / Ethics Committee (contact via XXX) for researchers who meet the criteria for access to confidential data.*<br><br>*The data underlying the results presented in the study are available*	Data are from the Swiss Mongolian pediatric Project whose authors may be contacted via its website: www.smopp.ch

from (include the name of the third party and contact information or URL).<br>• This text is appropriate if the data are owned by a third party and authors do not have permission to share the data.<br><br>* typeset	
Additional data availability information:	

NATIONAL CENTER FOR MATERNAL AND CHILD HEALTH

Ulaanbaatar, 23 January 2023

Submission of a manuscript to PLOS ONE "Comparison of the quality and interpretation of hip ultrasound screening examinations by basically trained nurses to junior physicians with no previous ultrasound experiences"

Dear Editor,

We are pleased to submit our manuscript for publication. We believe the paper is of particular interest to your readers.

Early diagnosis of developmental dysplasia of the hip (DDH) is the most important obligation for achieving the best outcome, using a simple, non-surgical method in the shortest possible time. The introduction of Graf's method of hip ultrasonography and more effective non-surgical treatment in Mongolia since 2010 changed the policy and time of diagnosis and preventive treatment in children with DDH, which in turn resulted in more prevented cases of childhood disabilities. Since 2019, the screening project has been expanded to major sub-provincial hospitals that are responsible for deliveries as a primary source of medical care of nomadic rural residents.

However, the sub-provincial hospitals face many significant and at times unique barriers when considering widespread adoption of newer innovations in health care such as hip ultrasound screening in newborns. One of the barrier is a lack of trained physicians capable of performing hip ultrasound (huge turnover within the doctor's teams, drainage of knowledge and ability etc) like other developing countries. Because resources are limited, physicians are not always available on an around-the-clock basis or when they are on remote emergency calls from rural remote areas. Thus this study was followed by a pilot expansion project to rural sub-provincial areas to compare the quality and accuracy of newborn hip ultrasound screening by basically trained nurses with no previous ultrasound experiences to basically trained junior physicians with no previous ultrasound experiences. The study provides evidence that after receiving a standard training in Graf's method of hip ultrasound, nurses are able to obtain standard hip sonograms according to Graf with equal quality as junior physicians.

The final version of the manuscript has been read and approved by all authors. It represents original work that has been published and that is not under consideration for publication elsewhere.

Thank you for considering our submission. We look forward to hearing from you.

Yours sincerely,

Munkhtulga Ulziibat MD, MSc

Tel: 976-99175523; Email: umunhtulga@gmail.com

Manuscript

Click here to access/download;Manuscript;Nurse_submited_final.pdf

Comparison of quality and interpretation of hip ultrasound screening examinations by basically trained nurses to junior physicians with no previous ultrasound experience

Munkhtulga Ulziibat[1, 2], Bayalag Munkhuu[2], Raoul Schmid[3], Corinne Wyder[4], Thomas Baumann[5], Stefan Essig[5]

[1]Department of Health Sciences and Medicine, University of Lucerne, Switzerland

[2]National Center for Maternal and Child Health, Ulaanbaatar, Mongolia

[3]Baarer Kinderarzt praxis, Baar, Switzerland

[4]Kinderärzte KurWerk, Burgdorf, Switzerland

[5]Center of Primary and Community Care, Department of Health Sciences and Medicine, University of Lucerne, Switzerland

Corresponding author

Correspondence to: Dr. Munkhtulga Ulziibat

Tel: 976-99175523

Email: umunhtulga@gmail.com

Abstract

Background: We are obliged to give babies the chance to profit from nationwide screening of developmental dysplasia of the hip in very rural areas of Mongolia, where trained physicians are not always available. This study aimed to compare the quality and interpretation of hip ultrasound screening examinations by nurses to junior physicians.

Methods: A group of 6 nurses and 6 junior physician volunteers with no previous ultrasound experience underwent Graf's standard training in hands-on practice. Newborns were examined before discharge from the hospital, according to the national guideline. Two standard documentation images of each hip were saved digitally. The groups were compared on the proportion of good quality of sonograms and correct interpretation. Two Swiss supervisors' agreed diagnosis according to Graf was considered the final reference for the study purposes.

Results: A total of 201 newborns were examined in the study, with a mean age of 1.3±0.8 days at examination. Junior physicians examined 100 newborns, while nurses examined 101 newborns. The study subjects of the two groups were well balanced for the distribution of baseline characteristics. The study observed no statistically significant difference in the quality of Graf's standard plane images between the providers. Eventually, 92.0% (92) of the physician group and 89.1% (90) of the nurse group were correctly diagnosed as "*Group A*" (Graf's Type 1 hip) or "*Non-Group A"* hips (p=0.484). The profession was not significantly associated with incorrect diagnosis in multivariate regression analysis. The most common errors among the groups were a missing lower limb, wrong measurement lines, and technical problems.

Conclusion: Our study provides evidence that after receiving standard training in Graf's hip ultrasound method, nurses can obtain standard hip sonograms according to Graf with equal quality as junior physicians. However, after basic training, regular quality control and refresher training are needed for nurses and physicians.

Keywords: hip ultrasound, Graf method, newborn screening, nurse study

Introduction

Early diagnos[illegible] of developmental dysplasia of the hip (DDH) [illegible] the most critical obligation for achieving the best outcome, using a simple, non-surgical method in the shortest possible time [1-4]. Delayed diagnosis results in losing the potential to remodel the acetabular roof, lengthening the treatment duration, and may cause lifelong disability [5, 6]. The hip ultrasound, according to Graf, which provides basic information about the biomechanical situation, is widely used in several countries as a gold standard of the primary tool in screening for DDH. Unlike other methods, it is standardized, examiner independent, easy to perform, and reproducible. The non-surgical treatment is usually based on the degree of severity of DDH [3, 7].

The introduction of Graf's method of hip ultrasonography and more effective non-surgical treatment in Mongolia since 2010 changed the policy and time of diagnosis and preventive treatment in children with DDH, resulting in more prevented cases of childhood disabilities [8, 9]. In 2017, the Mongolian Government approved the hip ultrasound screening as a nationwide screening program. This screening program aims to screen every newborn baby using hip ultrasound by Graf's method and to provide early prevention treatment to eliminate childhood disabilities due to DDH in the country. All major 7 maternity hospitals across Ulaanbaatar, the capital city, and all 21 provincial (aimag) general hospitals are equipped with necessary equipment and materials; and doctors trained in the method by Swiss-Mongolian Pediatric Project (SMOPP), the humanitarian aid project. Used Tübinger orthosis are collected in Switzerland and German-speaking countries and are being reuse[illegible]

Since 2019, the screening project has been expanded to major sub-provincial (soum) hospitals responsible for deliveries as a primary source of medical care for nomadic rural residents. However, the sub-provincial hospitals face many significant and unique barriers when considering the widespread adoption of newer innovations in health care, such as hip ultrasound screening in newborns. One barrier is a lack of trained physicians capable of performing hip ultrasounds (huge turnover within the doctor's teams, drainage of knowledge and ability, etc.) like in other developing countries [10]. Because of limited resources, physicians are not

always available on an around-the-clock or on remote emergency calls from rural areas. Therefore, we feel obliged to give the babies in the very rural areas the chance to profit from the nationwide screening. This study was followed by a pilot expansion project to rural sub-provincial areas to compare the quality and accuracy of newborn hip ultrasound screening by basically trained nurses with no previous ultrasound experiences to trained junior physicians with no previous ultrasound experiences.

Materials and methods

Study design and settings

A prospective cross-sectional study was conducted in 3 hospitals: a referral hospital in Ulaanbaatar, a provincial (aimag) general hospital, and a sub-provincial (soum) hospital in March-December 2021. The Ethics Review Committee of MOH, Mongolia, reviewed and approved the study protocol.

Study participants

A sample of 6 nurses and 6 junior physicians was self-selected from the nurse and junior physician volunteers in the selected 3 hospitals[illegible]ll consecutive newborns born at the 3 hospitals during the study period were ultrasonographically screened by six nurses (two from each hospital) and six junior physicians (two from each hospital). Exclusion criteria were severe or very severe conditions, congenital anomalies, and refusal of parents' written informed consent.

Preparatory phase

Nurse- and junior physician- volunteers who consented to participate in the study were trained on using the ultrasound machine through hands-on instruction. Two supervisors with extensive experience in hip sonography and the "AB[illegible]" system facilitated the training [11]. The nurses and junior physicians who have no prior knowledge or experience in hip sonography received training in basic hip sonography, with emphasis on ultrasound principles, macro, and sono-anatomy of the hip joint, obtaining standard images, and "ABCD" system, measurements,

tilting errors, pitfalls and counseling of parents. The standard week courses were organized in March in sub-provincial, August in provincial, and September 2021 in Ulaanbaatar hospitals.

Following the introductory course, the nurses and junior physicians completed a week of hip sonography screening and SMOPP's "HipScreen" platform practice in their hospitals, supervised by the supervisors who provided the training. It was intended that each nurse and junior physician undertake around 10-20 sonograms, focusing on gaining experience and competency in identifying children who require a referral (non-Group A) before testing their skills.

Data collection

Newborns were examined on a daily basis before discharge from the hospital (1st or 2nd days after birth) according to the national guideline [12]. Those who meet the selection criteria are recruited in the study, and the following procedures were done:

- counseling of mothers and brief information about DDH
- hip ultrasound examination
- information about the next steps according to hip ultrasound examination results.

All eligible newborns were examined using a GE LOGIQ series (C3) with a 7 to 8-MHz linear-array transduce[illegible] The examinations were performed and interpreted according to the national guideline based on the SMOPP's "ABCD" system [12], the modified Graf's system by the trained nurses or junior physicians depending on the days (odd days for nurses and even days for the junior physicians). Two standard documentation images of each hip were saved digitally using the HipScreen system. Follow-up control and management followed up the National guideline [13].

Two Swiss supervisors checked all the nurses' and junior physicians' hip ultrasound images. The two supervisors were blinded to the examiners. All

supervisors are internationally certified and have at least 10 years of experience in Graf's method of hip ultrasonography. Their final agreed diagnosis was considered the final reference for the study purposes. All supervisors ~~interpret~~ all examinations (nurses and junior physicians); if discordant, a discussion led to a ~~mutual result~~.

Outcome measurement

1. The following 4 indicators measured the quality of the newborn hip ultrasound images:

a) Anatomical identification is "correct" if all anatomical structures, according to Graf [3] are identified in the hip sonogram. If only one structure is missing, the sonogram is considered "incorrect".

b) The standard plane is "correct" if all 3 points according to Graf's criteria [3] (Lower limb of the bony ilium, Mid-portion of the acetabular roof, and Acetabular labrum) are visible in the hip sonogram. If only one structure is missing, the sonogram is considered "incorrect".

c) Three lines (baseline, bony, and cartilaginous roof lines) are measured "correct or incorrect" according to Graf's criteria [3].

d) The tilting was measured as "yes/no" depending on the absence/presence of any tilting errors according to Graf's criteria [3].

2. The correct interpretation: The screeners (nurses and junior physicians) were requested to comment on the "Group A" (Graf's Type 1 hip) or "*Non-Group A*" (Graf's non-Type 1 hip). Then it was checked against the reference interpretation performed by the Swiss experts. The interpretation was defined as "correct or incorrect".

Statistical analyses

Statistical analyses were performed using Stata 16 (Stata Corporation) software. Continuous variables were summarized as mean, standard deviation, and non-continuous variables as frequencies and percentages. A comparison of two groups

(nurses and junior physicians) was performed using the $\chi 2$ test, Fisher's exact test, and Student's t-tests depending on the variables with a significance level of 0.05 or less.

Univariate comparison of correct and incorrect diagnosis was performed using Fisher's exact test and the variables with a significance level of 0.2 or less were initially included in a logistic regression model. Those with highest likelihood ratio p-value were removed one-by-one based on backward elimination strategy until the model contained only significant variables with $p \leq 0.05$. In the main analysis, we used the child and not the hip sonogram (2 sonograms per hip or 4 hip sonograms per child) as the unit of analysis. If a child had hip sonograms with different qualities, we evaluated the child based on the worst quality.

Results

A group of 6 nurses and 6 junior physician volunteers with no previous ultrasound experience underwent Graf's standard training in hands-on practice and didactic instruction. The mean age of the junior physicians and nurses was 25±1.4 and 26.3±0.8 years, respectively. All were female.

A total of 201 newborns were examined in the study, with a male/female ratio of 118/83 and a mean age of 1.3±0.9 days at examination. Junior physicians examined 100 newborns, while nurses examined 101 newborns. The study subjects/newborns were well balanced for the distribution of baseline characteristics, such as mean age, sex, birth weight, and hospitals. (**Table 1**)

As shown in **Table 2**, of 100 newborns examined in the junior physician-performed group and 101 newborns examined in the nurse-performed group, the proportion of sufficient quality of hip sonograms was 92.0% and 89.1% (p=0.484), respectively. In the anatomical identification, 96 (96.0%) and 91 (90.1%) hip sonograms in the respective groups were correct (p=0.100). The incorrect standard plane was recorded in comparatively few proportions in both groups (3.0% in the junior physician's group vs. 8.9% in the nurse's group; p=0.077).

The proportion of measurement lines was 78.0% in the junior physician-performed group, while in the nurse-performed group, the proportion was 71.3% (p=0.274).

Eventually, 92.0% (90) of the junior group and 89.1% (90) of the nurse group (p=0.484) correctly diagnosed the "*Group A*" (Graf's Type 1 hip) or "*Non-Group A"* (Graf's non-Type 1 hip).

A common error among the groups was the tilting [illegible].0% in the junior group vs. 16.8% in the nurse's group, p=0.975), followed by the missing the lower limb (3.0% in the junior group vs. 7.9% in the nurse's group, p=0.125). (**Table 3**)

Univariate comparisons of demographic and hip ultrasound (Graf method) examination characteristics of "correct" and "incorrect" diagnosis are displayed in **Table 4**. The incorrect diagnosis was significantly more likely to be due to smaller birth weight of newborns, incorrect anatomical identification, incorrect measurement lines, missing lower limb and tilting errors.

Table 5 summarizes the independent variables for being incorrectly diagnosed using multivariate logistic regression analysis. The odds of incorrect diagnosis status was increased significantly in children having small birth weight (smaller for each 100 grams; OR=0.01, 95% CI 0.01, 05) and having incorrect anatomical identification (OR=1.1, 95% CI 0.1, 0.5) and incorrect lines (OR=20.4, 95% CI 5.2, 79.6), and missing lower limb (OR=2.2, 95% CI 1.3, 3.2). Nurse performed hip examinations were likely to be incorrect (OR=1.3, 95% CI 0.4, 4.4), but it was not statistically significant (p=0.595).

Discussion

The study provides evidence that nurses could be a practical alternative where suitable hip ultrasound-trained doctors are unavailable. The "Graf's standard image" quality success proportion of 92.0% and 89.1% were observed for junior physicians' and nurses' groups, respectively. Furthermore, the study revealed similar "Group A" or "Non-Group A" hips' correct identification rates in both groups. The profession was not significantly associated with incorrect diagnosis in

multivariate regression analysis.The common error among the groups was missing the lower limb.

In developing countries implementing and sustaining any newborn screening, including newborn hip ultrasound, is challenging due to potential political instability, lack of trained human resources and less developed public health systems, etc [13]. Over the past decade, the humanitarian Swiss-Mongolian Pediatric Project SMOPP, commissioned by the MOH in Mongolia, has established a newborn ultrasound screening and early conservative treatment program for developmental dysplasia of the hip [9]. The obligation to implement a DDH ultrasound screening program, especially in a country with limited resources, requires plausible, simple structures and straightforward algorithms. These requirements led to two paradigms in the implementation of the SMOPP: a) application of the Graf's ultrasound method [3] as early as possible in the newborn period in a simplified form with only 4 intervention groups [11], b) uniform, simple, cost-effective outpatient treatment of all DDH severity levels with a flexion-abduction device performed by the parents under pediatric surveillance [11].

In Mongolia, the Graf's method is used in a simplified modified ABCD adaptation [11], which combines the Graf types into intervention groups according to therapeutic aspects (A = normal; B = physiologically immature, worthy of control; C = DDH, in need of immediate therapy through flexion-abduction-orthosis; D = dislocated). Knowing the high maturation potential in the first 3 months of life [14], the strategy must be implemented as early as possible after birth. The feasibility of the concept in Mongolia was proven by a cohort study and subsequent statistical analyses [9]. All cases (n=107) of DDH discovered in newborns were cured using the Tübingen orthosis [*15]. In addition, the hip ultrasound screening has been well accepted by local newborn hip ultrasound screeners (e.g., neonatologists and pediatricians) even though the screening was comparatively new to the providers. Nevertheless, the screening implementation has not been smooth, especially in prominent sub-provincial hospitals* physicians are not always available when they are on remote emergency calls from remote rural areas.

The need for a possible alternative approach to screen every newborn baby for DDH by ultrasound is obvious when trained doctors are not available, especially in rural areas. To address the challenge of detecting “non-Group A” (Graf’s non-Type 1 hip) cases before discharge from maternity hospital (usually, after birth, newborns stay 1-2 days), it might be wise to train nurses since nurses are a more stable group in terms of mobility or changing workplace in the country [16] This is especially crucial in rural hospitals where nomadic residents receive maternity and health services. Thus our study aimed to evaluate the feasibility of nurses without prior experience in ultrasonography to obtain the standard plane images of newborn hips. The study observed no statistically significant difference in the quality of Graf’s standard plane images between the providers: junior physicians and nurse[illegible] Also in our study the profession was not significantly associated with incorrect diagnosis in multivariate regression analysis.

Although the role of nurse-led hip ultrasonography is insufficiently studied, Professor Graf, the founder of the hip ultrasound method, repeatedly mentioned that the nurse-performed hip ultrasound has good accuracy. There is a study that aimed to evaluate healthcare workers’ ability to classify ultrasound images into a Graf system [17]. The study team consisted of 3 physicians and 4 nurses at the infant health care system, with no previous ultrasound experience in the Netherlands. After theoretical and practical training, seven nurses and physicians of the participating infant health centers reported their findings on hip sonograms of 80 children. This was repeated five months later. From the two evaluation moments, the intra-observer agreement and the inter-observer agreement were determined. Based on the study results, the authors concluded that the inter- and intra-observer agreement is comparable to similar studies in which the participants had a professional background in ultrasound examination. The level of agreement of the trainees in the perspective of the screening process was considered sufficient [17]. However, this study addressed only the intra- and inter-observer variability in reading a sonogram. Therefore, the findings of this study and our results cannot be compared.

On the other hand, nurses are key players in the newborn screening program, and their role is crucial in increasing newborn screening coverage [18] Considered front liners, nurses are usually the first contact with parents in a primary care setting or health facility, allowing them to advocate and educate parents on newborn screening. Besides these advantages recently, several studies have shown that appropriately trained nurses can perform some ultrasound scans [19-24] Although the role of nurse-led hip ultrasonography is poorly studied, it has been described as helpful in the placement of central[21] and peripheral intravenous lines [19] in patients with difficult access, for the focused assessment in emergency situations [23, 25-26] and patients with urologic [22, 27], obstetric [22, 28], and cardiac diseases [20].

In the present study, common errors were missing the lower limb in both groups and subsequent measurement bias that led to the wrong diagnosis. It is mandatory to check the usefulness of a hip sonogram according to the Graf method: a) to identify the lower limb of the os ilium, b) the precise middle plain of the acetabulum roof, and c) the labrum (lower limb – plain - labrum); and errors of tilting must be excluded. Such errors may eventually lead to incorrect procedures that may cause unnecessary over or under-treatment for babies without a quality control system [29,30] However, in the study, a quality control system allowed reliable review of the diagnosis and treatment of DDH and continuous education of nurses and junior doctors.

The current study has some potential limitations. 1) The generalizability of this study into the whole nurses' community of different levels of care might be limited because only six nurses and six physicians performed the examinations. However, we tried to have a delegation from different levels of care hospitals, e.g., from referral, provincial and sub-provincial hospitals. Concerning the study's limitations, the nurses' or junior physicians' characteristics might result in variations of skills related to image acquisition and interpretation which could affect the quality of the hip ultrasound scan. 2) The nurses' and junior physicians' evaluations of newborns' hips were not validated against a reference method such as computed tomography

or magnetic resonance imaging. However, Graf's method of hip ultrasound is standardized, it accepts only a standard plane cut of the acetabulum, and hence it is also reproducible. Our study focused on evaluating whether nurses and junior physicians can obtain the correct plane of the hip sonograms and identify "non-Group A or Graf's non-Type 1). The study did not address the intra- and inter-observer agreement regarding hip sonograms. Nevertheless, the quality and interpretation of the nurse- and junior physician-performed hip ultrasound images were compared.

Conclusion

Our study provides evidence that nurses can obtain standard hip sonograms with equal quality as junior physicians after receiving standard training in Graf's hip ultrasound method. However, after a basic training, regular quality control and refresher training are compulsory for both nurses and physicians.

Acknowledgements

We would like to thank all families, nurses and physicians who participated in this study. We would also like to thank our colleagues who provided expert advice and are members of the Swiss Mongolian Pediatric project and the Mongolian Society of Developmental Dysplasia of the Hip Prevention.

References

1. Joseph Gerard O'Beirne, Konstantinos Chlapoutakis, Sattar Alshryda, et al. international consensus meeting on evaluation of developmental dysplasia of the hip. Ultraschall in Med 2019; 40:454-464.

2. Thallinger C, Pospischill R, Ganger R, et al. Long term results of a nationwide general ultrasound screening system for developmental disorders of the hip: the Austrian hip screening program. *J Child Orthop.* 2014; 8:3–10. doi: 10.1007/s11832-014-0555-6.

3. Graf R, Baumgartner F, Lercher K, Scott S, Benaroya A. Hip Sonography. Diagnosis and Management of Infant Hip Dysplasia. 2006. Springer ISBN 3-540-30957-8

4. Kotlarsky P, Haber R, Bialik V, Eidelman M. Developmental dysplasia of the hip: What has changed in the last 20 years? World J Orthop. 2015; 6(11):886-901. doi: 10.5312/wjo.v6.i11.886. PMID: 26716085; PMCID: PMC4686436.

5. Brdar R, Petronic I, Nikolic D, Lukac M, Cirovic D, Blagojevic T. Walking quality after surgical treatment of developmental dysplasia of the hip in children. Acta Orthop Belg 2013; 79(1):60-63.

6. Fujii M, Nakashima Y, Jingushi S, et al. Intraarticular findings in symptomatic developmental dysplasia of the hip. J Pediatr Orthop 2009; 29: 9–13.

7. Graf R. The diagnosis of congenital hip-joint dislocation by the ultrasonic compound treatment. *Arch Orthop Trauma Surg.* 1980; 97:117–133. doi:10.1007/BF00450934

8. Munkhuu B, Essig S, Renchinnyam E, Schmid R, Wilhelm C, et al. Incidence and Treatment of Developmental Hip Dysplasia in Mongolia: A Prospective Cohort Study. PLoS ONE 2013; 8(10): e79427. doi:10.1371/journal.pone.0079427.

9. Ulziibat M, Munkhuu B, Schmid R, Baumann T, Essig S. Implementation of a nationwide universal ultrasound screening programme for developmental

dysplasia of the neonatal hip in Mongolia. *J Child Orthop* 2020; 14:273-280. DOI: 10.1302/1863-2548.14.200029

10. World Health Organization. Regional Office for Europe, European Observatory on Health Systems and Policies, Bolormaa, T, Natsagdorj, Ts, Tumurbat, B. et al. Mongolia: health system review. World Health Organization. Regional Office for Europe. 2007. https://apps.who.int/iris/handle/10665/107845

11. Baumann T, Schmid R, Essig S, et al ABCD OF DDH. Zumsteg Druck AG. 2017 https://www.thieme-connect.com/products/ejournals/html/10.1055/s-0043-110690.

12. Ministry of Health Mongolia. National guideline of newborn ultrasound screening and management for the developmental dysplasia of the hip. Ministry of Health Mongolia. Ulaanbaatar. 2021

13. Therrell BL,Padilla CD. Barriers to implementing sustainable national newborn screening in developing health systems. International Journal of Pediatrics and Adolescent Medicine. 2014; 1(2):49-60.

14. Tschauner C, Klapsch W, Baumgarthner A, Graf R. Maturation curve of the ultrasonographic alpha angle according to Graf's untreated hip joint in the first year of life. Z Orthop Ihre Grenzgeb 1994; 132:502-504.

15. Baumann T, Munkhuu B, Chuluunbaatar B, Schmid R. 4-year follow-up of ultrasound-based diagnosis and non-surgical treatment of developmental dysplasia of the hip in Mongolia: a prospective cohort study. Ultraschall in der Medizin. 2016; 37(S 01) DOI: 10.1055/s-0036-1587724. https://www.thieme-connect.com/products/ ejournals/abstract/10.1055/ s-0036-1587724

16. World Health Organization. Regional Office for Europe, European Observatory on Health Systems and Policies, Bolormaa, T, Natsagdorj, Ts, Tumurbat, B. et al. Mongolia: health system review. World Health Organization. Regional Office for Europe. 2007. https://apps.who.int/iris/handle/10665/107845

17. Ramwadhdoebe S, Sakkers RJ, Uiterwaal CS, Boere-Boonekamp MM, Beek FJ. Evaluation of a training program for general ultrasound screening for developmental dysplasia of the hip in preventive child health care. Pediatr Radiol. 2010; 40(10):1634-9. doi: 10.1007/s00247-010-1689-4. Epub 2010 Jun 12. PMID: 20544189.

18. DeLuca J, Zanni KL, Bonhomme N, Kemper AR. Implications of newborn screening for nurses. J Nurs Scholarsh. 2013; 45(1):25-33. doi: 10.1111/jnu.12005. Epub 2013 Jan 31. PMID: 23368429.

19. Stokowski G, Steele D, Wilson D. The use of ultrasound to improve practice and reduce complication rates in peripherally inserted central catheter insertions: final report of investigation. J Infus Nurs 2009; 32:145–55.

20. Colquhoun SM, Carapetis JR, Kado JH, Reeves BM, Remenyi B, May W, Wilson NJ, Steer AC. Pilot study of nurse-led rheumatic heart disease echocardiography screening in Fiji - a novel approach in a resource-poor setting. Cardiol Young 2013; 23:546–52.

21. Weiner SG, Sarff AR, Esener DE, Shroff SD, Budhram GR, Switkowski KM, Mostofi MB, Barus RW, Coute RA, Darvish AH. Single-operator ultrasound-guided intravenous line placement by Emergency Nurses reduces the need for physician intervention in patients with difficult-to-establish intravenous access. J Emerg Med 2013; 44: 653–60.

22. Paez A, Ju_arez N, Mar_ın M, Del Vigo S, Redondo E, Dur_an M. Concordance analysis of ultrasonographic diagnoses by urology nurses. Arch Esp Urol 2012; 65: 872–8.

23. Bowra J, Forrest-Horder S, Caldwell E, Cox M, D’Amours SK. Validation of nurse-performed FAST ultrasound. Injury 2010; 41: 484–7.

24. Menihan CA. Limited obstetric ultrasound in nursing practice. J Obstet Gynecol Neonatal Nurs. 2000; 29(3):325-30. doi: 10.1111/j.1552-6909.2000.tb02054.x. PMID: 10839581.

25. Bloom BA, Gibbons RC. Focused Assessment with Sonography for Trauma. In: StatPearls [Internet]. Treasure Island (FL): Stat Pearls Publishing; 2022. https://www.ncbi.nlm.nih.gov/books/NBK470479/

26. Wayne Varndell, Michelle Topacio, Carmel Hagness, Hayley Lemon, Deborah Tracy. Nurse-performed focused ultrasound in the emergency department: A systematic review. Literature review 2018; 21(4):121-130. DOI:https://doi.org/10.1016/j.auec.2018.09.30.

27. Nandwana SB, Walls DG, Ibraheem O, Murphy F, Tridandapani S, Cox K. Image-Guided Nontargeted Renal Biopsies Performed by Radiology-Trained Nurse Practitioners: A Safe Practice Model. J Am Coll Radiol. 2016; 13(7):819-21.

28. Marilyn Stringer, Susan R. Miesnik, Linda P. Brown, Lynne Menei, George A. Macones. Limited Obstetric Ultrasound Examinations: Competency and Cost. Clinical research 2003; 32:307-312. DOI:https://doi.org/10.1177/0884217503253452.

29. Tschauner, C, Matthissen, H. Hip Sonography with Graf-method in Newborns: Checklists help to avoid mistakes. OUB 2012; 1:7–8.

30. Graf R, Mohajer M, Plattner F. Hip sonography update. Quality management, catastrophes - tips and tricks. Med Ultrason 2013; 15:299-303.

Table 1. Newborn characteristics

Characteristics	All<br>N=201<br>n(%)	Junior physician-performed N=100<br>n(%)	Nurse-performed<br>N=101<br>n(%)	P value
Mean age at hip ultrasound examination, days (mean±sd)	1.3±0.9	1.3±0.8	1.3±0.9	0.2877*
Sex				0.711**
Male	118 (58.7)	60 (60.0)	58 (57.4)	
Female	83 (41.3)	40 (40.0)	43 (42.6)	
Hospital				0.139**
Referral	98 (48.8)	55 (55.0)	43 (42.6)	
Provincial and sub-provincial	103 (51.2)	45 (45.0)	58 (57.4)	
Mean birth weight, kg (mean±sd)	3.2±0.2	3.2±0.2	3.2±0.2	0.410*

**t-test, **Chi^2 test*

Table 2. The quality of the newborn hip ultrasound images

Characteristics	Junior physician-performed N=100 n(%)	Nurse-performed N=101 n(%)	P value*
Quality of hip sonograms			0.484
Sufficient	92 (92.0)	90 (89.1)	
Insufficient	8 (8.0)	11 (10.9)	
Anatomical identification		0.245	0.100
Correct	96 (96.0)	91 (90.1)	
Incorrect	4 (4.0)	10 (9.9)	
Standard plane			[illegible].077
Correct	97 (97.0)	92 (91.1)	
Incorrect	3 (3.0)	9 (8.9)	
Measurement lines			0.274
Correct	78 (78.0)	72 (71.3)	
Incorrect	22 (22.0)	29 (28.7)	
Diagnosis			0.484
Correct	92 (92.0)	90 (89.1)	
Incorrect	8 (8.0)	11 (10.9)	

**Chi*^{2} *test*

Table 3. Common errors of the study groups

Mistakes	Junior physician-performed N=100 n(%)	Nurse-performed N=101 n(%)	P value*
Tilting			0.975
Yes	15 (15.0)	19 (18.8)	
No	85 (85.0)	82 (81.2)	
Missing lower limb			0.125
Yes	3 (3.0)	8 (7.9)	
No	97 (97.0)	93 (92.1)	

**Fisher exact test*

Table 4. Demographic and hip ultrasound examination characteristics of correct and incorrect diagnosis

Characteristics	Correct diagnosis N=182	Incorrect diagnosis N=9	p-value
Age of newborns (CI)	1.3 ± 0.9 (1.2 - 1.5)	1.2 ± 0.7 (0.7 - 1.5)	0.439
Mean birth weight (CI)	3.2 ±0.2 (3.2 - 3.3)	3.1 ± 0.2 (2.9 - 3.2)	0.0123
Hospital			0.275
UB	91 (50.0)	7 (36.8)	
rural	91(50.0)	12 (63.2)	
Performed by			0.484
junior physician	92 (50.6)	8 (42.1)	
nurse	90 (49.4)	11 (57.9)	
Anatomical identification			<0.0001
correct	182 (100.0)	5 (26.3)	
incorrect	0	14 (73.7)	
Standard plain cut			<0.0001
correct	182 (100.0)	7 (36.8)	
incorrect	0	12 (63.2)	
Measurement lines			<0.0001
correct	148 (81.3)	2 (10.5)	

incorrect	34 (18.7)	17 (89.5)	
Tilting errors			0.002
yes	26 (14.3)	8 (42.1)	
no	156 (85.7)	11 (57.9)	
Missing lower limb			<0.0001
yes	0	11 (57.9)	
no	182	8 (42.1)	

*****Fisher exact test

Table 5. Significant factors related to incorrect diagnosis from final logistic regression model

Characteristics	Odds ratio	95% confidence interval	p value
Small birth weight of newborn (per 100 grams)	0.01	0.01-0.5	0.021
Incorrect anatomical identification	1.1	0.1-0.5	<0.001
Incorrect lines	20.4	5.2-79.6	<0.001
No lower limb	2.2	1.3-3.2	<0.001
Nurse performed	1.3	0.4-4.4	0.595
