# Supplementary material for: Comparison of quality and interpretation of newborn ultrasound screening examinations for developmental dysplasia of the hip by basically trained nurses and junior physicians with no previous ultrasound experience
Source: PLoS One. 2024 Apr 18;19(4):e0300753. doi: 10.1371/journal.pone.0300753 (PMC11025947; doi:10.1371/journal.pone.0300753)
Supplement: Attachment — Submitted filename: PONE-D-23-02980_R1_AFreviewer.pdf [file pone.0300753.s006.pdf]

# PLOS ONE

## Comparison of quality and interpretation of hip ultrasound screening examinations by basically trained nurses to junior physicians with no previous ultrasound experience

--Manuscript Draft--

| | |
|---|---|
| **Manuscript Number:** | PONE-D-23-02980R1 |
| **Article Type:** | Research Article |
| **Full Title:** | Comparison of quality and interpretation of hip ultrasound screening examinations by basically trained nurses to junior physicians with no previous ultrasound experience |
| **Short Title:** | Comparison of quality of hip ultrasound screening examinations by nurses to junior physicians |
| **Corresponding Author:** | Munkhtulga Ulziibat, M.D<br>National Center for Maternal and Child Health of Mongolia<br>Ulaanbaatar, MONGOLIA |
| **Keywords:** | hip ultrasound, Graf method, newborn screening, nurse study |
| **Abstract:** | Abstract<br>Background: Because resources are limited, adequately trained physicians are not always available, especially in remote rural areas. We are obliged to give babies in very rural areas of Mongolia the chance to profit from nationwide screening of developmental dysplasia of the hip. Thus, this study aimed to compare the quality and accuracy of newborn hip ultrasound screening examinations by nurses to junior physicians with no previous experience after standard training.<br>Methods: A group of 6 nurses and 6 junior physician volunteers with no previous ultrasound experience underwent Graf's standard training in hands-on practice and didactic instruction with prospective follow-up in urban and rural hospitals. Newborns were examined before discharge from the hospital, according to the national guideline. Two standard documentation images of each hip were saved digitally. The groups were compared on the proportion of good quality of sonograms and correct interpretation. Two Swiss supervisors' agreed diagnosis according to Graf was considered the final reference for the study purposes.<br>Results: A total of 201 newborns were examined in the study, with a mean age of 1.3±0.8 days at examination. Junior physicians examined 100 newborns, while nurses examined 101 newborns. The study subjects of the two groups were well balanced for the distribution of baseline characteristics, such as mean age, sex, birth weight, and hospitals.<br>The study observed no statistically significant difference in the quality of Graf's standard plane images between the providers: junior physicians and nurses (92.0% and 89.1%; p=0.484). Eventually, 92.0% (92) of the physician group and 89.1% (90) of the nurse group were correctly diagnosed as "Group A" (Graf's Type 1 hip) or "Non-Group A" hips (p=0.484). The most common errors among the groups were a missing lower limb, wrong measurement lines, and technical problems.<br>Conclusion: Our study provides evidence that after receiving standard training in Graf's hip ultrasound method, nurses can obtain standard hip sonograms according to Graf with equal quality as junior physicians. However, after basic training, regular quality control and refresher training are needed for nurses and physicians. |
| **Order of Authors:** | Munkhtulga Ulziibat, M.D |
| | Bayalag Munkhuu, PhD |
| | Raoul Schmid |
| | Corinne Wyder |
| | Thomas Baumann |
| | Stefan Essig, PhD |
| **Response to Reviewers:** | Editor Comments<br>The manuscript has been evaluated by five reviewers, and their comments are available below. The reviewers have raised a number of concerns throughout your |

| | |
|---|---|
| | manuscript that need attention. They recommend additional background information in the Introduction to better introduce the topic. They also feel the Methods section may benefit from further clarification, particularly as it pertains to the education of the trainees, and the inclusion of statistical power calculations which may validate conclusions drawn from your study. It is also suggested to include additional images which may enhance presentation for the reader. Could you please revise the manuscript to carefully address the concerns raised?<br>Response: Thank you and the reviewers very much for the opportunity to submit a revision of our manuscript. We added additional background information, information on the education of the trainees and statistical power calculations, as well as images. Below, we respond to each point raised by the reviewers. If we can provide any additional information or make any other changes, please do not hesitate to let us know.<br>The manuscript has important value for dissemination, given the needs at the primary care level in many countries. The study and results are well designed and explained. However, perhaps from the language and writing parts there are several unclear expressions that may confuse the readers. Thus, I have made several suggestions so that you and authors can review, revise, correct and then re-submit. Thank you.<br>Response: Thank you for your support. We are not sure which suggestions came from you as the comments of you and the reviewers were submitted in three parts (text in the e-mail, PDF and Word file). We respond to each point from all three parts below and carefully address the concerns raised. |
| **Additional Information:** | |
| **Question** | **Response** |
| **Financial Disclosure**<br><br>Enter a financial disclosure statement that describes the sources of funding for the work included in this submission. Review the submission guidelines for detailed requirements. View published research articles from *PLOS ONE* for specific examples.<br><br>This statement is required for submission and **will appear in the published article** if the submission is accepted. Please make sure it is accurate. | The first author received doctoral training support from the Swiss Mongolian Pediatric Project (SMOPP) http://www.sipp.swiss/ and the Swiss Association of Pediatric<br>Ultrasound (SVUPP). Moreover, ultrasound machines and examination cradles were provided<br>by SMOPP free of charge under the governmental national screening program. The funders had no role in study design, data collection and analysis, decision to publish, or preparation of the manuscript. |

*Powered by Editorial Manager® and ProduXion Manager® from Aries Systems Corporation*

| | |
|---|---|
| **Unfunded studies**<br>Enter: *The author(s) received no specific funding for this work.*<br><br>**Funded studies**<br>Enter a statement with the following details:<br>• Initials of the authors who received each award<br>• Grant numbers awarded to each author<br>• The full name of each funder<br>• URL of each funder website<br>• Did the sponsors or funders play any role in the study design, data collection and analysis, decision to publish, or preparation of the manuscript?<br>• **NO** - Include this sentence at the end of your statement: *The funders had no role in study design, data collection and analysis, decision to publish, or preparation of the manuscript.*<br>• **YES** - Specify the role(s) played.<br><br>* typeset | |
| **Competing Interests**<br><br>Use the instructions below to enter a competing interest statement for this submission. On behalf of all authors, disclose any competing interests that could be perceived to bias this work—acknowledging all financial support and any other relevant financial or non-financial competing interests.<br><br>This statement is required for submission and **will appear in the published article** if the submission is accepted. Please make sure it is accurate and that any funding sources listed in your Funding Information later in the submission form are also declared in your Financial Disclosure statement.<br><br>View published research articles from *PLOS ONE* for specific examples. | The authors have declared that no competing interests exist. |

| | |
|---|---|
| **NO authors have competing interests**<br><br>Enter: *The authors have declared that no competing interests exist.*<br><br>**Authors with competing interests**<br><br>Enter competing interest details beginning with this statement:<br><br>*I have read the journal's policy and the authors of this manuscript have the following competing interests: [insert competing interests here]*<br><br>* typeset | |
| **Ethics Statement**<br><br>Enter an ethics statement for this submission. This statement is required if the study involved:<br><br>• Human participants<br>• Human specimens or tissue<br>• Vertebrate animals or cephalopods<br>• Vertebrate embryos or tissues<br>• Field research<br><br>Write "N/A" if the submission does not require an ethics statement.<br><br>General guidance is provided below. Consult the submission guidelines for detailed instructions. **Make sure that all information entered here is included in the Methods section of the manuscript.** | Mongolian Ethical Committee, commissioned by the Ministry of Health |

Powered by Editorial Manager® and ProduXion Manager® from Aries Systems Corporation

| | |
|---|---|
| **Format for specific study types**<br><br>**Human Subject Research (involving human participants and/or tissue)**<br>• Give the name of the institutional review board or ethics committee that approved the study<br>• Include the approval number and/or a statement indicating approval of this research<br>• Indicate the form of consent obtained (written/oral) or the reason that consent was not obtained (e.g. the data were analyzed anonymously)<br><br>**Animal Research (involving vertebrate animals, embryos or tissues)**<br>• Provide the name of the Institutional Animal Care and Use Committee (IACUC) or other relevant ethics board that reviewed the study protocol, and indicate whether they approved this research or granted a formal waiver of ethical approval<br>• Include an approval number if one was obtained<br>• If the study involved *non-human primates*, add *additional details* about animal welfare and steps taken to ameliorate suffering<br>• If anesthesia, euthanasia, or any kind of animal sacrifice is part of the study, include briefly which substances and/or methods were applied<br><br>**Field Research**<br><br>Include the following details if this study involves the collection of plant, animal, or other materials from a natural setting:<br>• Field permit number<br>• Name of the institution or relevant body that granted permission | |
| **Data Availability**<br><br>Authors are required to make all data underlying the findings described fully available, without restriction, and from the time of publication. PLOS allows rare exceptions to address legal and ethical concerns. See the PLOS Data Policy and FAQ for detailed information. | Yes - all data are fully available without restriction |

*Powered by Editorial Manager® and ProduXion Manager® from Aries Systems Corporation*

| | |
|---|---|
| A Data Availability Statement describing where the data can be found is required at submission. Your answers to this question constitute the Data Availability Statement and **will be published in the article**, if accepted.<br><br>**Important:** Stating 'data available on request from the author' is not sufficient. If your data are only available upon request, select 'No' for the first question and explain your exceptional situation in the text box.<br><br>Do the authors confirm that all data underlying the findings described in their manuscript are fully available without restriction? | |
| **Describe where the data may be found in full sentences. If you are copying our sample text, replace any instances of XXX with the appropriate details.**<br><br>• If the data are **held or will be held in a public repository**, include URLs, accession numbers or DOIs. If this information will only be available after acceptance, indicate this by ticking the box below. For example: *All XXX files are available from the XXX database (accession number(s) XXX, XXX.*).<br>• If the data are all contained **within the manuscript and/or Supporting Information files**, enter the following: *All relevant data are within the manuscript and its Supporting Information files.*<br>• If neither of these applies but you are able to provide **details of access elsewhere**, with or without limitations, please do so. For example:<br><br>*Data cannot be shared publicly because of [XXX]. Data are available from the XXX Institutional Data Access / Ethics Committee (contact via XXX) for researchers who meet the criteria for access to confidential data.*<br><br>*The data underlying the results presented in the study are available from (include the name of the third party* | Data are from the Swiss Mongolian pediatric Project whose authors may be contacted via its website: www.smopp.ch |

| | |
|---|---|
| *and contact information or URL).*<br>• This text is appropriate if the data are owned by a third party and authors do not have permission to share the data.<br><br>* typeset | |
| Additional data availability information: | |

Manuscript Click here to access/download;Manuscript;Manuscript.docx

# Comparison of quality and interpretation of newborn ultrasound screening examinations for developmental dysplasia of the hip by basically trained nurses and junior physicians with no previous ultrasound experience

*Munkhtulga Ulziibat[1, 2], Bayalag Munkhuu[2], Raoul Schmid[3], Corinne Wyder[4], Thomas Baumann[5], Stefan Essig[5]*

[1]*Department of Health Sciences and Medicine, University of Lucerne, Switzerland*

[2]*National Center for Maternal and Child Health, Ulaanbaatar, Mongolia*

[3]*Baarer Kinderarzt praxis, Baar, Switzerland*

[4]*Kinderärzte KurWerk, Burgdorf, Switzerland*

[5]*Center of Primary and Community Care, Department of Health Sciences and Medicine, University of Lucerne, Switzerland*

**Corresponding author**

Correspondence to: Dr. Munkhtulga Ulziibat

Tel: 976-99175523

Email: umunhtulga@gmail.com

## Abstract

**Background:** We are obliged to give babies the chance to profit from a nationwide screening of developmental dysplasia of the hip in very rural areas of Mongolia, where trained physicians are scarce. This study aimed to compare the quality and interpretation of hip ultrasound screening examinations performed by nurses and junior physicians.

**Methods:** A group of 6 nurses and 6 junior physician volunteers with no previous ultrasound experience underwent Graf's standard training in hands-on practice. Newborns were examined before discharge from the hospital, according to the national guideline. Two standard documentation images of each hip were saved digitally. The groups were compared on the proportion of good quality of sonograms and correct interpretation. Two Swiss supervisors' agreed diagnosis according to Graf was considered the final reference for the study purposes.

**Results:** A total of 201 newborns (402 hips or 804 sonograms) were examined in the study, with a mean age of 1.3±0.8 days at examination. Junior physicians examined 100 newborns (200 hips or 400 sonograms), while nurses examined 101 newborns (202 hips or 404 sonograms). The study subjects of the two groups were well balanced for the distribution of baseline characteristics. The study observed no statistically significant difference in the quality of Graf's standard plane images between the providers. Eventually, 92.0% (92) of the physician group and 89.1% (90) of the nurse group were correctly diagnosed as "Group A" (Graf's Type 1 hip) or "Non-Group A" hips (p=0.484). The most common errors among the groups were a missing lower limb, wrong measurement lines, and technical problems.

**Conclusion:** Our study provides evidence that while there might be a trend of slightly more technical mistakes in the nurse group, the overall diagnosis accuracy is similar to junior physicians after receiving standard training in Graf's hip ultrasound method. However, after basic training, regular quality control is a must and all participants

should receive refresher trainings. More specifically, nurses need training in the identification of anatomical structures.

**Keywords:** hip ultrasound, Graf method, newborn screening, nurse study

## Introduction

Developmental dysplasia of the hip (DDH) is one of the most common disorders of the osteoarticular system with public health priorities in otherwise healthy children. The reported incidence of DDH are quite variable depending on detection methods, ages and diagnostic criteria [1, 2]. Mongolian studies reported a 1-2% incidence of DDH among neonates by ultrasound with Graf's technique [3], an incidence comparable to that in European neonates [4, 5]. DDH is a multifactorial disorder. Contributing factors for the development of DDH are genetic and non-genetic factors [6, 7]. Early diagnosis of DDH in neonates is the most critical obligation for achieving the best outcome, using a simple, non-surgical method in the shortest possible time [8-11]. Delayed diagnosis results in losing the potential to remodel the acetabular roof, lengthening the treatment duration, and may cause lifelong disability [12, 13].

The hip ultrasound, according to Graf, which provides basic information about the biomechanical situation, is widely used in several countries as a gold standard of the primary tool in screening for DDH. Unlike other methods, it is standardized, examiner independent, easy to perform, and reproducible. The non-surgical treatment is usually based on the degree of severity of DDH [3, 14]. Graf's method has been shown to be sensitive and specific for the early diagnosis of DDH [15]. Studies have also shown that ultrasonographic screening by Graf is cost-effective [16]. The method cuts through the center of the acetabulum (standard plain) and the hip is evaluated by measuring two angles formed by three lines drawn from three landmarks (lower limb or the bottom of the acetabulum, the acetabular labrum and the lateral edge of the acetabulum). Subsequently, the bony roof angle (alpha angle) and the cartilage roof angle (beta angle) are measured and the hip joint is classified (**Figure 1**).

The introduction of Graf's method of hip ultrasonography and more effective non-surgical treatment in Mongolia since 2010 changed the policy and time of diagnosis and

preventive treatment in children with DDH, resulting in more prevented cases of childhood disabilities [4, 5]. In 2017, the Mongolian Government approved the hip ultrasound screening as a nationwide screening program. This screening program aims to screen every newborn baby using hip ultrasound by Graf's method and to provide early prevention treatment to eliminate childhood disabilities due to DDH in the country. All major 7 maternity hospitals across Ulaanbaatar, the capital city, and all 21 provincial (aimag) general hospitals are equipped with the necessary equipment and materials; and doctors trained in the method by the Swiss-Mongolian Pediatric Project (SMOPP), a humanitarian aid project. Used Tübingen braces are collected in Switzerland and German-speaking countries and are being reused as a way to help correct the skeletal system.

In Mongolia, a web-based, password-protected platform is used for quality control purpose. The tool enables screeners in all hospitals to upload digital images and annotations exported from the ultrasound machines. Four images (two per hip side; one of them with measured alpha and beta angles according to Graf), a unique identification number, age, sex and diagnosis are required for all examinations. After the upload, quality is controlled by a local expert (a doctor with profound knowledge of the Graf method and at least three years of experience of hip ultrasound and treatment at a provincial hospital). This allowes continuous and reliable reviews of all examinations. In addition, Mongolian supervisors (doctors with profound knowledge of the Graf method and more than 10 years of experience of hip ultrasound and treatment in UB) check examinations from all hospitals and promptly send comments to the experts and screeners.

Since 2019, the screening project has been expanded to major sub-provincial (soum) hospitals responsible for deliveries as a primary source of medical care for nomadic rural residents. However, the sub-provincial hospitals face many significant and unique

barriers when considering the widespread adoption of newer innovations in health care, such as hip ultrasound screening in newborns. One barrier is a lack of trained physicians capable of performing hip ultrasounds (huge turnover within the doctors' teams, loss of trained doctors due to high internal and international migrations etc.) like in other developing countries [17]. Therefore, we felt obliged to give the babies in very rural areas the chance to profit from the nationwide screening program. We searched for other possibilities to examine all newborn babies of nomadic families. This study was performed after 3 years of piloting the expansion to rural sub-provincial areas. We aimed to compare the quality and interpretation of newborn hip ultrasound examinations (Graf's Type 1 versus "Non-type 1" hips) between basically trained nurses with no previous ultrasound experiences and trained junior physicians with no previous ultrasound experience.

## Materials and methods

### *Study design and settings*

A prospective cross-sectional study was conducted in 3 hospitals: a referral hospital in Ulaanbaatar, a provincial (aimag) general hospital, and a sub-provincial (soum) hospital from March to December 2021. The Ethics Review Committee of the Ministry of Health of Mongolia (MOH), reviewed and approved the study protocol.

### *Study participants*

A sample of 6 nurses and 6 junior physicians were nurse and junior physician volunteers in the selected hospitals. The only selection criterion was that they had no previous knowledge or experience in ultrasound. Other criteria were considered obsolete as study participants' volunteering led to a selection of persons that would also start ultrasound examinations outside the framework of the study. The six nurses were all certified with bachelor degree (4 years of undergraduate training) from Nursing school of Mongolian

National University of Medical Science and had 3-4 years (4 nurses- 3 years and 2 nurses- 4 years) of experience. The six junior physicians were first-year pediatric resident doctors and all received undergraduate training in medicine (six years) from Mongolian National University of Medical Science. All junior physicians had 1-2 years of clinical experience in primary care.

All consecutive newborns born at the 3 hospitals during the study period were ultrasonographically screened by six nurses (two from each hospital) and six junior physicians (two from each hospital). Exclusion criteria were severe or very severe conditions, congenital anomalies, and refusal of parents' written informed consent.

*Preparatory phase*

Nurse- and junior physician- volunteers who consented to participate in the study were trained on using the ultrasound machine through hands-on instruction. Two supervisors with extensive experience in hip sonography and the "ABCD" system facilitated the training [5]. The "ABCD" system is a diagnostic and therapeutic framework based on Graf's technique, SMOPP realized that age-dependent Graf subtypes are irrelevant in the screened neonates in the first week of life. Moreover, it was not practical for the screeners at maternity hospitals to learn all of the different diagnostic subtypes and subsequent treatments outlined by Graf because Graf Type 2c, D and 3 hips are treated in the same way (i.e. flexion-abduction braces). Therefore, the original Graf system required some adjustments into a simpler "ABCD" system. This system is entirely based on the method described by Graf and translates his initial differentiation into four groups: Group A includes Graf Type 1; Group B includes Graf Type 2a; Group C includes Graf Types 2c, D and 3; and Group D includes Graf Type 4 (**Table 1**).

The nurses and junior physicians received training in basic hip sonography, with emphasis on ultrasound principles, macro, and sono-anatomy of the hip joint, obtaining standard images, and "ABCD" system, measurements, tilting errors, pitfalls and

counseling of parents. The standard week courses (15 hours including practical works) were organized in March in sub-provincial, August in provincial, and September 2021 in Ulaanbaatar hospitals. There was no pre-training test since all participants had no previous knowledge or experience in Graf's method of hip ultrasound examinations. However, a post-training test was performed and included questions on theoretical and practical (interpretation and handling) knowledge. The average score of the test was not significantly different between the two groups.

Following the standard introductory course, the nurses and junior physicians completed a week of hip sonography screening and SMOPP's "HipScreen" quality control online platform practice in their hospitals, supervised by the supervisors who provided the training. Each nurse and junior physician undertook 10 newborns' hip sonograms (2 sonograms of right and left hips totaling 80 sonograms), focusing on gaining experience and competency in identifying children who require a referral (non-Group A hips) before testing their skills.

*Data collection*

Newborns were examined on a daily basis before discharge from the hospital ($1^{st}$ or $2^{nd}$ days after birth) according to the national guideline [19]. Those who met the selection criteria were recruited in the study, and the following procedures were done:

- counseling of mothers and brief information about DDH
- hip ultrasound examination
- information about the next steps according to hip ultrasound examination results.

All eligible newborns were examined using a GE LOGIQ series (C3) ultrasound machine with a 7 to 8-MHz linear-array transducer. The examinations were performed and interpreted according to the national guideline based on the SMOPP's "ABCD"

system [18], the modified Graf's system by the trained nurses or junior physicians depending on the days (odd days for nurses and even days for the junior physicians). Two standard documentation images of each hip were saved digitally using the HipScreen system. Follow-up control and management followed the National guideline [19].

Two Swiss supervisors checked all the nurses' and junior physicians' hip ultrasound images. The two supervisors were blinded to the nurses and doctors. All supervisors are internationally certified and have at least 10 years of experience in Graf's method of hip ultrasonography. Their final agreed diagnosis was considered the final reference for the study purposes. All supervisors interpreted all examinations (nurses and junior physicians); if discordant, a discussion led to a consensual agreement.

***Outcome measurement***

1. The following 4 indicators measured the quality of the newborn hip ultrasound images:

a) Anatomical identification is "correct" if all anatomical structures, according to Graf [3, 12] are identified in the hip sonogram. If at least one structure is missing, the sonogram is considered "incorrect".

b) The standard plane is "correct" if all 3 points according to Graf's criteria [3, 12] (Lower limb of the bony ilium, Mid-portion of the acetabular roof, and Acetabular labrum) are visible in the hip sonogram. If at least one structure is missing, the sonogram is considered "incorrect".

c) Three lines (baseline, bony, and cartilaginous roof lines) are measured "correct" or "incorrect" according to Graf's criteria [3, 12].

d) The tilting was measured as "yes" or "no" depending on the absence/presence of any tilting errors according to Graf's criteria [3, 12]. In order to get a standard plain

sonogram the transducer should be placed vertically on the hip joint. Tilted positions of the transducer can lead to significant examinations errors (usually a mechanical transducer guide prevents tilting errors by reducing the degrees of variability).

2. The correct interpretation: The screeners (nurses and junior physicians) were requested to comment on the “Group A” (Graf’s Type 1 hip) or “Non-Group A” (Graf’s non-Type 1 hip). Then it was checked against the reference interpretation performed by the Swiss experts. The interpretation was defined as “correct” or “incorrect”.

**Statistical analyses**

Statistical analyses were performed using Stata 16 (Stata Corporation) software. Continuous variables were summarized as mean, standard deviation, and non-continuous variables as frequencies and percentages. A comparison of two groups (nurses and junior physicians) was performed using the $\chi 2$ test, Fisher’s exact test, and Student’s t-tests depending on the variables, with a significance level of 0.05 or less.

Univariate comparison of correct and incorrect diagnosis was performed using Fisher’s exact test. In the main analysis, we used the child and not the hip sonogram (2 sonograms per hip or 4 hip sonograms per child) as the unit of analysis. If a child had hip sonograms with different qualities, we evaluated the child based on the worst quality.

Power calculations indicate that we need to include at least 92 subjects in each group based on the assumptions that 1) the expected proportion of insufficient quality of hip ultrasound of the junior physician group is 10%, 2) the expected proportion of insufficient quality of hip ultrasound of the nurse group is 25%, 3) the alpha level is 0.05, and 4) the required power is 80%.

**Results**

A group of 6 nurses and 6 junior physician volunteers with no previous ultrasound experience underwent Graf’s standard training in hands-on practice and didactic

instruction. The mean age of the junior physicians and nurses was 25±1.4 and 26.3±0.8 years, respectively. All were female.

A total of 201 newborns (402 hips or 804 hip sonograms) were examined in the study, with a male/female ratio of 118/83 and a mean age of 1.3±0.9 days at examination. Junior physicians examined 100 newborns (200 hips or 400 hip sonograms), while nurses examined 101 newborns (202 hips or 404 hip sonograms). The study subjects/newborns were well balanced for the distribution of baseline characteristics, such as mean age, sex, birth weight, and hospitals. (**Table 2**)

As shown in **Figure 2**, of 100 newborns examined in the junior physician-performed group and 101 newborns examined in the nurse-performed group, the proportion of sufficient quality of hip sonograms was 92.0% and 89.1% (p=0.484), respectively. In the anatomical identification, 96 (96.0%) and 91 (90.1%) newborns' hip sonograms in the respective groups were correct (p=0.100). The incorrect standard plane was recorded in comparatively few proportions in both groups (3.0% in the junior physician's group vs. 8.9% in the nurse's group; p=0.077).

The proportion of measurement lines was 78.0% in the junior physician-performed group, while in the nurse-performed group, the proportion was 71.3% (p=0.274).

Eventually, 92.0% (90) of the junior group and 89.1% (90) of the nurse group (p=0.484) correctly diagnosed the "Group A" (Graf's Type 1 hip) or "Non-Group A" (Graf's non-Type 1 hip). (**Figure 2**)

A common error among the groups was the tilting (17.0% in the junior group vs. 16.8% in the nurse's group, p=0.975), followed by the missing the lower limb (3.0% in the junior group vs. 7.9% in the nurse's group, p=0.125). (**Figure 2**)

Univariate comparisons of demographic and hip ultrasound (Graf method) examination characteristics of "correct" and "incorrect" diagnosis are displayed in **Table 3**. The

incorrect diagnosis was significantly more likely to be due to smaller birth weight of newborns (p=0.012), incorrect anatomical identification (p<0.0001), incorrect measurement lines (p<0.0001), missing lower limb and tilting errors (p<0.002).

## Discussion

The study provides evidence that nurses could be a practical alternative where suitable hip ultrasound-trained doctors are unavailable. The "Graf's standard image" quality success proportion of 92.0% and 89.1% were observed for junior physicians' and nurses' groups, respectively. Furthermore, the study revealed similar "Group A" or "Non-Group A" hips' correct identification rates in both groups. The most common error among the groups were tilting and missing the lower limb.

In developing countries, implementing and sustaining any newborn screening, including newborn hip ultrasound, is challenging due to potential political instability, lack of trained human resources and less developed public health systems, etc [20]. Over the past decade, the humanitarian Swiss-Mongolian Pediatric Project - SMOPP, commissioned by the MOH in Mongolia, has established a newborn ultrasound screening and early conservative treatment program for developmental dysplasia of the hip [4, 5]. The obligation to implement a DDH ultrasound screening program, especially in a country with limited resources, requires plausible, simple structures and straightforward algorithms. These requirements led to two paradigms in the implementation of the SMOPP: a) application of the Graf's ultrasound method [3, 12] as early as possible in the newborn period in a simplified form with only 4 intervention groups [18], and b) uniform, simple, cost-effective outpatient treatment of all DDH severity levels with a flexion-abduction device performed by the parents under pediatric surveillance [3, 12, 18].

In Mongolia, the Graf's method is used in a simplified modified ABCD adaptation [18], which combines the Graf types into intervention groups according to therapeutic aspects (A = normal; B = physiologically immature, worthy of control; C = DDH, in need of immediate therapy through flexion-abduction-braces; D = dislocated). Knowing the high maturation potential in the first 3 months of life [21], the strategy must be implemented as early as possible after birth. The feasibility of the concept in Mongolia was proven by a cohort study [4] and subsequent statistical analyses [5]. All cases (n=107) of DDH discovered in newborns were cured using the Tübingen braces [22]. In addition, the hip ultrasound screening has been well accepted by local newborn hip ultrasound screeners (e.g., neonatologists and pediatricians) even though the screening was comparatively new to the providers. Nevertheless, the screening implementation has not been smooth, especially in prominent sub-provincial hospitals because physicians are not always available when they are on remote emergency calls from remote rural areas.

The need for a possible alternative approach to screen every newborn baby for DDH by ultrasound is obvious when trained doctors are not available, especially in rural areas. To address the challenge of detecting "non-Group A" (Graf's non-Type 1 hip) cases before discharge from maternity hospital (usually, after birth, newborns stay 1-2 days), it might be wise to train nurses since nurses are a more stable group in terms of mobility or changing workplace in the country [23]. This is especially crucial in rural hospitals where nomadic residents receive maternity and health services. Thus our study aimed to evaluate the feasibility of nurses without prior experience in ultrasonography to obtain the standard plane images of newborn hips. The study observed no statistically significant difference in the quality of Graf's standard plane images between the providers: junior physicians and nurses. Nevertheless, the study suggests that the nurses might need more training to correctly identify anatomical structures.

Although the role of nurse-led hip ultrasonography is insufficiently studied, Professor Graf, the founder of the hip ultrasound method, repeatedly mentioned that the nurse-performed hip ultrasound has good accuracy. There is a study that aimed to evaluate healthcare workers' ability to classify ultrasound images into a Graf system [24]. The study team consisted of 3 physicians and 4 nurses at the infant health care system, with no previous ultrasound experience in the Netherlands. After theoretical and practical training, seven nurses and physicians of the participating infant health centers reported their findings on hip sonograms of 80 children. This was repeated five months later. From the two evaluation moments, the intra-observer agreement and the inter-observer agreement were determined. Based on the study results, the authors concluded that the inter- and intra-observer agreement is comparable to similar studies in which the participants had a professional background in ultrasound examination. The level of agreement of the trainees in the perspective of the screening process was considered sufficient [24]. However, this study addressed only the intra- and inter-observer variability in reading a sonogram. Therefore, the findings of this study and our results cannot be compared.

On the other hand, nurses are key players in the newborn screening program, and their role is crucial in increasing newborn screening coverage [25] Considered front liners, nurses are usually the first contact with parents in a primary care setting or health facility, allowing them to advocate and educate parents on newborn screening. Besides these advantages recently, several studies have shown that appropriately trained nurses can perform some ultrasound scans [26-31]. Although the role of nurse-led hip ultrasonography is poorly studied, it has been described as helpful in the placement of central [26] and peripheral intravenous lines [26, 28] in patients with difficult access, for the focused assessment in emergency situations [30, 32, 33] and patients with urologic [29, 34], obstetric [31, 35], and cardiac diseases [27].

In the present study, common errors were missing the lower limb in both groups and subsequent measurement bias that led to the wrong diagnosis. It is mandatory to check the usefulness of a hip sonogram according to the Graf method: a) to identify the lower limb of the os ilium, b) the precise middle plain of the acetabulum roof, and c) the labrum (lower limb – plain - labrum); and errors of tilting must be excluded. Such errors may eventually lead to incorrect procedures that may cause unnecessary over or under-treatment for babies without a quality control system [36, 37]. However, in the study, a quality control system allowed reliable review of the diagnosis and treatment of DDH and continuous education of nurses and junior doctors.

The current study has some potential limitations. 1) The generalizability of this study into the whole nurses' community of different levels of care might be limited because only six nurses and six physicians performed the examinations. However, we tried to have a delegation from different levels of care hospitals, e.g., from referral, provincial and sub-provincial hospitals. Concerning the study's limitations, the nurses' or junior physicians' characteristics might result in variations of skills related to image acquisition and interpretation which could affect the quality of the hip ultrasound scan. 2) The nurses' and junior physicians' evaluations of newborns' hips were not validated against a reference method such as computerized tomography or magnetic resonance imaging. However, Graf's method of hip ultrasound is standardized, it accepts only a standard plane cut of the acetabulum, and hence it is also reproducible. Our study focused on evaluating whether nurses and junior physicians can obtain the correct plane of the hip sonograms and identify "non-Group A" or "Graf's non-Type 1" hips. The study did not address the intra- and inter-observer agreement regarding hip sonograms. Nevertheless, the quality and interpretation of the nurse- and junior physician-performed hip ultrasound images were compared.

## Conclusion

Our study provides evidence that while there might be a trend of slightly more technical mistakes in the nurse group, the overall diagnostic accuracy is similar to junior physicians after receiving standard training in Graf's hip ultrasound method. However, after a basic standard training, regular quality control is a must and all participants should receive refresher trainings. More specifically, nurses need training in the identification of anatomical structures.


## Acknowledgements

We would like to thank all families, nurses and physicians who participated in this study. We would also like to thank our colleagues who provided expert advice and are members of the Swiss Mongolian Pediatric Project and the Mongolian Society of Developmental Dysplasia of the Hip Prevention.

## References


1. Loder RT, Skopelja EN. The epidemiology and demographics of hip dysplasia. ISRN Orthop. 2011; 2011:238607. https://doi.org/10.5402/2011/ 238607

2. Shipman SA, Helfand M, Moyer VA, Yawn BP. Screening for developmental dysplasia of the hip: a systematic literature review for the US preventive services task force. Pediatrics. 2006; 117(3):557–576. https://doi.org/10. 1542/peds

3. Graf, R. Fundamentals of sonographic diagnosis of infant hip dysplasia. J. Pediatr. Orthop. 1984, 4:735–740.

4. Munkhuu B, Essig S, Renchinnyam E, Schmid R, Wilhelm C, et al. Incidence and treatment of developmental hip dysplasia in Mongolia: a prospective cohort study. PLoS One. 2013; 8(10):e79427. https://doi.org/10.1371/journ al.pone.0079427

5. Ulziibat M, Munkhuu B, Schmid R, Baumann T, Essig S. Implementation of a nationwide universal ultrasound screening programme for developmental dysplasia of the neonatal hip in Mongolia. J Child Orthop. 2020; 14. https://doi.org/10.1302/1863-2548.14.200029.

6. Carter C, Wilkinson J. Genetic and environmental factors in the etiology of congenital dislocation of the hip. Clin Orthop Relat Res. 1964; 33:119–128.

7. Ulziibat M, Munkhuu B, Bataa AE, Schmid R, Baumann T, Essig S. Traditional Mongolian swaddling and developmental dysplasia of the hip: a randomized controlled trial. BMC Pediatr. 2021 Oct 13;21(1):450. doi: 10.1186/s12887-021-02910-x. PMID: 34641800; PMCID: PMC8513275.

8. 1.Joseph Gerard O'Beirne, Konstantinos Chlapoutakis, Sattar Alshryda, et al. international consensus meeting on evaluation of developmental dysplasia of the hip. Ultraschall in Med 2019; 40:454-464.

9. Thallinger C, Pospischill R, Ganger R, et al. Long term results of a nationwide general ultrasound screening system for developmental disorders of the hip: the Austrian hip screening program. J Child Orthop. 2014; 8:3–10. doi: 10.1007/s11832-014-0555-6.

10. Graf R, Baumgartner F, Lercher K, Scott S, Benaroya A. Hip Sonography. Diagnosis and Management of Infant Hip Dysplasia. 2006. Springer ISBN 3-540-30957-8

11. Kotlarsky P, Haber R, Bialik V, Eidelman M. Developmental dysplasia of the hip: What has changed in the last 20 years? World J Orthop. 2015; 6(11):886-901. doi: 10.5312/wjo.v6.i11.886. PMID: 26716085; PMCID: PMC4686436.

12. Brdar R, Petronic I, Nikolic D, Lukac M, Cirovic D, Blagojevic T. Walking quality after surgical treatment of developmental dysplasia of the hip in children. Acta Orthop Belg 2013; 79(1):60-63.

13. Fujii M, Nakashima Y, Jingushi S, et al. Intraarticular findings in symptomatic developmental dysplasia of the hip. J Pediatr Orthop 2009; 29: 9–13.

14. Graf R. The diagnosis of congenital hip-joint dislocation by the ultrasonic compound treatment. Arch Orthop Trauma Surg. 1980; 97:117–133. doi:10.1007/BF00450934

15. O’Beirne JG, Chlapoutakis K, Alshryda S, et al. International interdisciplinary consensus meeting on evaluation of developmental dysplasia of the hip. Ultraschall Med 2019; 40:454-464.

16. Thaler M, Biedermann R, Lair J, Krismer M, Landauer F. Cost-effectiveness of universal ultrasound screening compared with clinical examination alone in the diagnosis and treatment of neonatal hip dysplasia in Austria. J Bone Joint Surg [Br] 2011; 93-B:1126-1130.

17. World Health Organization. Regional Office for Europe, European Observatory on Health Systems and Policies, Bolormaa, T, Natsagdorj, Ts, Tumurbat, B. et al.

Mongolia: health system review. World Health Organization. Regional Office for Europe. 2007. https://apps.who.int/iris/handle/10665/107845

18. Baumann T, Schmid R, Essig S, et al ABCD OF DDH. Zumsteg Druck AG. 2017 https://www.thieme-connect.com/products/ejournals/html/10.1055/s-0043-110690.

19. Ministry of Health, Mongolia. National guideline of newborn ultrasound screening and management for the developmental dysplasia of the hip. Ministry of Health, Mongolia. Ulaanbaatar. 2021

20. Therrell BL,Padilla CD. Barriers to implementing sustainable national newborn screening in developing health systems. International Journal of Pediatrics and Adolescent Medicine. 2014; 1(2):49-60.

21. Tschauner C, Klapsch W, Baumgarthner A, Graf R. Maturation curve of the ultrasonographic alpha angle according to Graf's untreated hip joint in the first year of life. Z Orthop Ihre Grenzgeb 1994; 132:502-504.

22. Baumann T, Munkhuu B, Chuluunbaatar B, Schmid R. 4-year follow-up of ultrasound-based diagnosis and non-surgical treatment of developmental dysplasia of the hip in Mongolia: a prospective cohort study. Ultraschall in der Medizin. 2016; 37(S 01) DOI: 10.1055/s-0036-1587724. https://www.thieme-connect.com/products/ejournals/abstract/10.1055/ s-0036-1587724

23. World Health Organization. Regional Office for Europe, European Observatory on Health Systems and Policies, Bolormaa, T, Natsagdorj, Ts, Tumurbat, B. et al. Mongolia: health system review. World Health Organization. Regional Office for Europe. 2007. https://apps.who.int/iris/handle/10665/107845

24. Ramwadhdoebe S, Sakkers RJ, Uiterwaal CS, Boere-Boonekamp MM, Beek FJ. Evaluation of a training program for general ultrasound screening for developmental

dysplasia of the hip in preventive child health care. Pediatr Radiol. 2010; 40:1634-1639. doi: 10.1007/s00247-010-1689-4. Epub 2010 Jun 12. PMID: 20544189.

25. DeLuca J, Zanni KL, Bonhomme N, Kemper AR. Implications of newborn screening for nurses. J Nurs Scholarsh. 2013; 45:25-33. doi: 10.1111/jnu.12005. Epub 2013 Jan 31. PMID: 23368429.

26. Stokowski G, Steele D, Wilson D. The use of ultrasound to improve practice and reduce complication rates in peripherally inserted central catheter insertions: final report of investigation. J Infus Nurs 2009; 32:145–155.

27. Colquhoun SM, Carapetis JR, Kado JH, Reeves BM, Remenyi B, May W, Wilson NJ, Steer AC. Pilot study of nurse-led rheumatic heart disease echocardiography screening in Fiji - a novel approach in a resource-poor setting. Cardiol Young 2013; 23:546–552.

28. Weiner SG, Sarff AR, Esener DE, Shroff SD, Budhram GR, Switkowski KM, Mostofi MB, Barus RW, Coute RA, Darvish AH. Single-operator ultrasound-guided intravenous line placement by Emergency Nurses reduces the need for physician intervention in patients with difficult-to-establish intravenous access. J Emerg Med 2013; 44: 653–660.

29. Paez A, Ju_arez N, Mar_ın M, Del Vigo S, Redondo E, Dur_an M. Concordance analysis of ultrasonographic diagnoses by urology nurses. Arch Esp Urol 2012; 65: 872–878.

30. Bowra J, Forrest-Horder S, Caldwell E, Cox M, D’Amours SK. Validation of nurse-performed FAST ultrasound. Injury 2010; 41: 484–487.

31. Menihan CA. Limited obstetric ultrasound in nursing practice. J Obstet Gynecol Neonatal Nurs. 2000; 29:325-330. doi: 10.1111/j.1552-6909.2000.tb02054.x. PMID: 10839581.

32. Bloom BA, Gibbons RC. Focused Assessment with Sonography for Trauma. In: StatPearls [Internet]. Treasure Island (FL): Stat Pearls Publishing; 2022. https://www.ncbi.nlm.nih.gov/books/NBK470479/

33. Wayne Varndell, Michelle Topacio, Carmel Hagness, Hayley Lemon, Deborah Tracy. Nurse-performed focused ultrasound in the emergency department: A systematic review. Literature review 2018; 21(4):121-130. DOI:https://doi.org/10.1016/j.auec.2018.09.30.

34. Nandwana SB, Walls DG, Ibraheem O, Murphy F, Tridandapani S, Cox K. Image-Guided Nontargeted Renal Biopsies Performed by Radiology-Trained Nurse Practitioners: A Safe Practice Model. J Am Coll Radiol. 2016; 13(7):819-821.

35. Marilyn Stringer, Susan R. Miesnik, Linda P. Brown, Lynne Menei, George A. Macones. Limited Obstetric Ultrasound Examinations: Competency and Cost. Clinical research 2003; 32:307-312. DOI:https://doi.org/10.1177/0884217503253452.

36. Tschauner, C, Matthissen, H. Hip Sonography with Graf-method in Newborns: Checklists help to avoid mistakes. OUB 2012; 1:7–8.

37. Graf R, Mohajer M, Plattner F. Hip sonography update. Quality management, catastrophes - tips and tricks. Med Ultrason 2013; 15:299-303.

**Figure 1. A hip sonogram**.

A. Three landmarks of the standard plain. 1. Standard cut (red arrow), 2. Lower limb or the bottom of the acetabulum (yellow circle), 3. Acetabular labrum and the lateral edge of the acetabulum (blue circle).

B. Hip is evaluated by measuring two angles formed by three lines drawn from three landmarks. 1. Basic line (red), 2. Bony roof line (blue), 3. Cartilaginous roof line (yellow)

**Figure 2. Hip ultrasound examination performance of the nurses's and junior physicians' groups**

**Table 1. Comparison of Graf types and ABCD groups for hip ultrasound**

* Group B hips were defined as angle alpha minus the age in weeks = between 50°-60°

| Graf type | Graf angle $\alpha$ (°) | Graf angle $\beta$ (°) | Graf therapy | ABCD group | ABCD angle $\alpha$ (°) | ABCD therapy |
|---|---|---|---|---|---|---|
| **1a** | > 60 | < 55 | None | **A** | > 60 | None |
| **1b** | | > 55 | None | | | |
| **2a** | 50–59 | | Control | **B** | > 50 ≤ 60* | Control |
| **2a+** | | | Control | | | |
| **2a-** | | | Spreading device/Pavlik | **C** | < 50 | Tübingen braces |
| **2b** | | | | | | |
| **2c stable** | 43–49 | < 77 | | | | |
| **2c unstable** | | | Plaster | | | |
| **D** | | > 77 | | | | |
| **3a** | < 43 | | Extension | | | |
| **3b** | | | | | | |
| **4** | | | Operation | **D** | - | Tübingen braces/Operation |

**Table 2. Newborn characteristics**

| **Characteristics** | **All N=201 n(%)** | **Junior physician-performed N=100 n(%)** | **Nurse-performed N=101 n(%)** | **P value** |
|---|---|---|---|---|
| Mean age at hip ultrasound examination, days (mean±sd) | 1.3±0.9 | 1.3±0.8 | 1.3±0.9 | 0.2877* |
| **Sex** | | | | 0.711** |
| Male | 118 (58.7) | 60 (60.0) | 58 (57.4) | |
| Female | 83 (41.3) | 40 (40.0) | 43 (42.6) | |
| Hospital | | | | 0.078** |
| Referral | 98 (48.8) | 55 (55.0) | 43 (42.6) | |
| Provincial and sub-provincial | 103 (51.2) | 45 (45.0) | 58 (57.4) | |
| Mean birth weight, kg (mean±sd) | 3.2±0.2 | 3.2±0.2 | 3.2±0.2 | 0.820* |

**t-test, **Chi*$^2$ *test*

**Table 3. ~~Univaraite~~ analysis of demographic and hip ultrasound examination characteristics of correct and incorrect diagnosis**

| Characteristics | Correct diagnosis N=182 | Incorrect diagnosis N=9 | p-value |
|---|---|---|---|
| Age of newborns (CI) | 1.3 ± 0.9 (1.2 - 1.5) | 1.2 ± 0.7 (0.7 - 1.5) | 0.439 |
| Mean birth weight (CI) | 3.2 ±0.2 (3.2 - 3.3) | 3.1 ± 0.2 (2.9 - 3.2) | 0.0123 |
| Hospital | | | 0.275 |
| UB | 91 (50.0) | 7 (36.8) | |
| rural | 91(50.0) | 12 (63.2) | |
| Performed by | | | 0.484 |
| junior physician | 92 (50.6) | 8 (42.1) | |
| nurse | 90 (49.4) | 11 (57.9) | |
| Anatomical identification | | | <0.0001 |
| correct | 182 (100.0) | 5 (26.3) | |
| incorrect | 0 | 14 (73.7) | |
| Standard plain cut | | | <0.0001 |
| correct | 182 (100.0) | 7 (36.8) | |
| incorrect | 0 | 12 (63.2) | |
| Measurement lines | | | <0.0001 |

| | | | |
|---|---|---|---|
| correct | 148 (81.3) | 2 (10.5) | |
| incorrect | 34 (18.7) | 17 (89.5) | |
| Tilting errors | | | 0.002 |
| yes | 26 (14.3) | 8 (42.1) | |
| no | 156 (85.7) | 11 (57.9) | |
| Lower limb missing | | | <0.0001 |
| yes | 0 | 11 (57.9) | |
| no | 182 | 8 (42.1) | |

******Fisher exact*

Figure 1A

Click here to access/download;Figure;Figure 1A.jpg

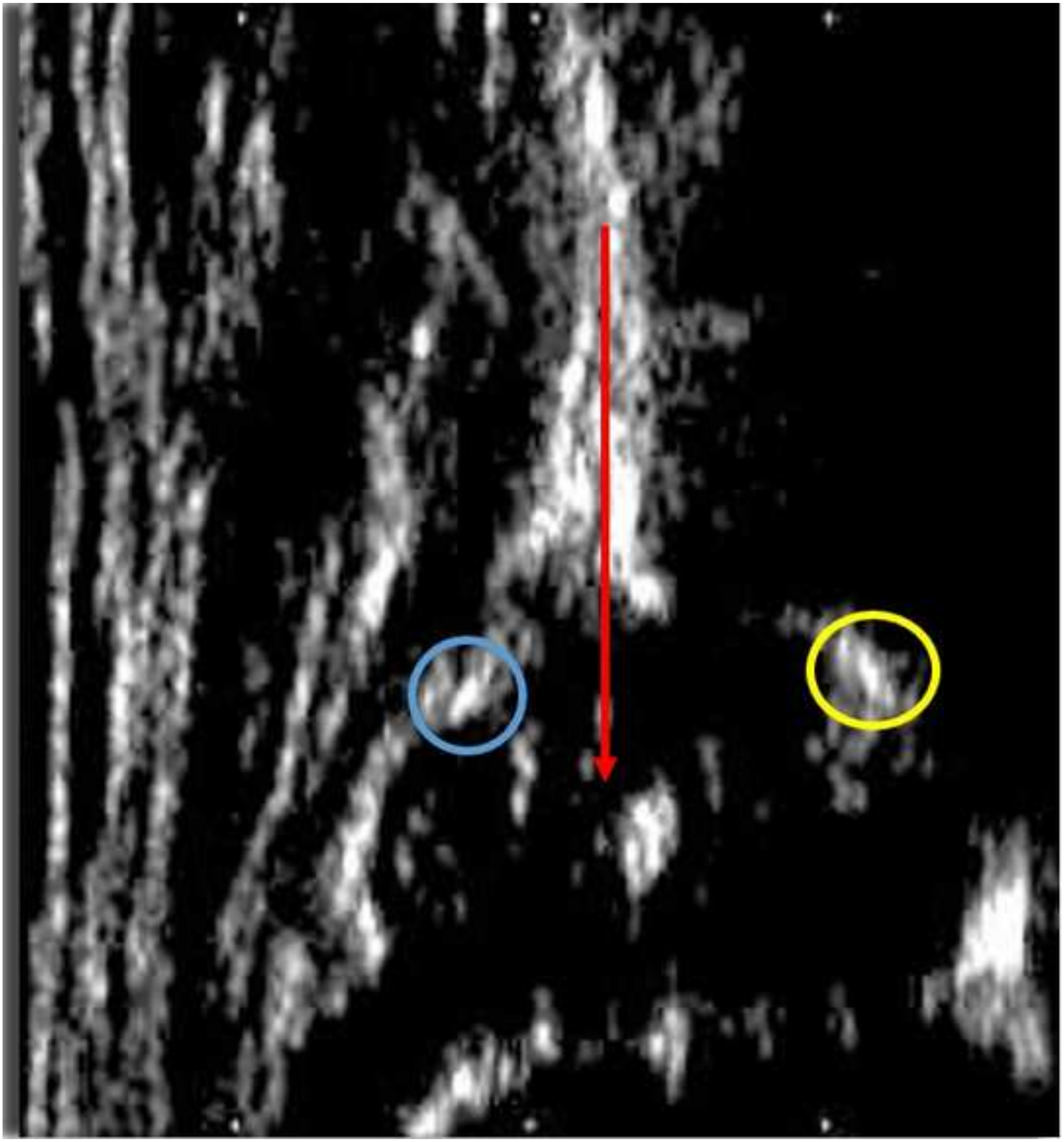

Figure 1B

Click here to access/download;Figure;Figure 1B.jpg

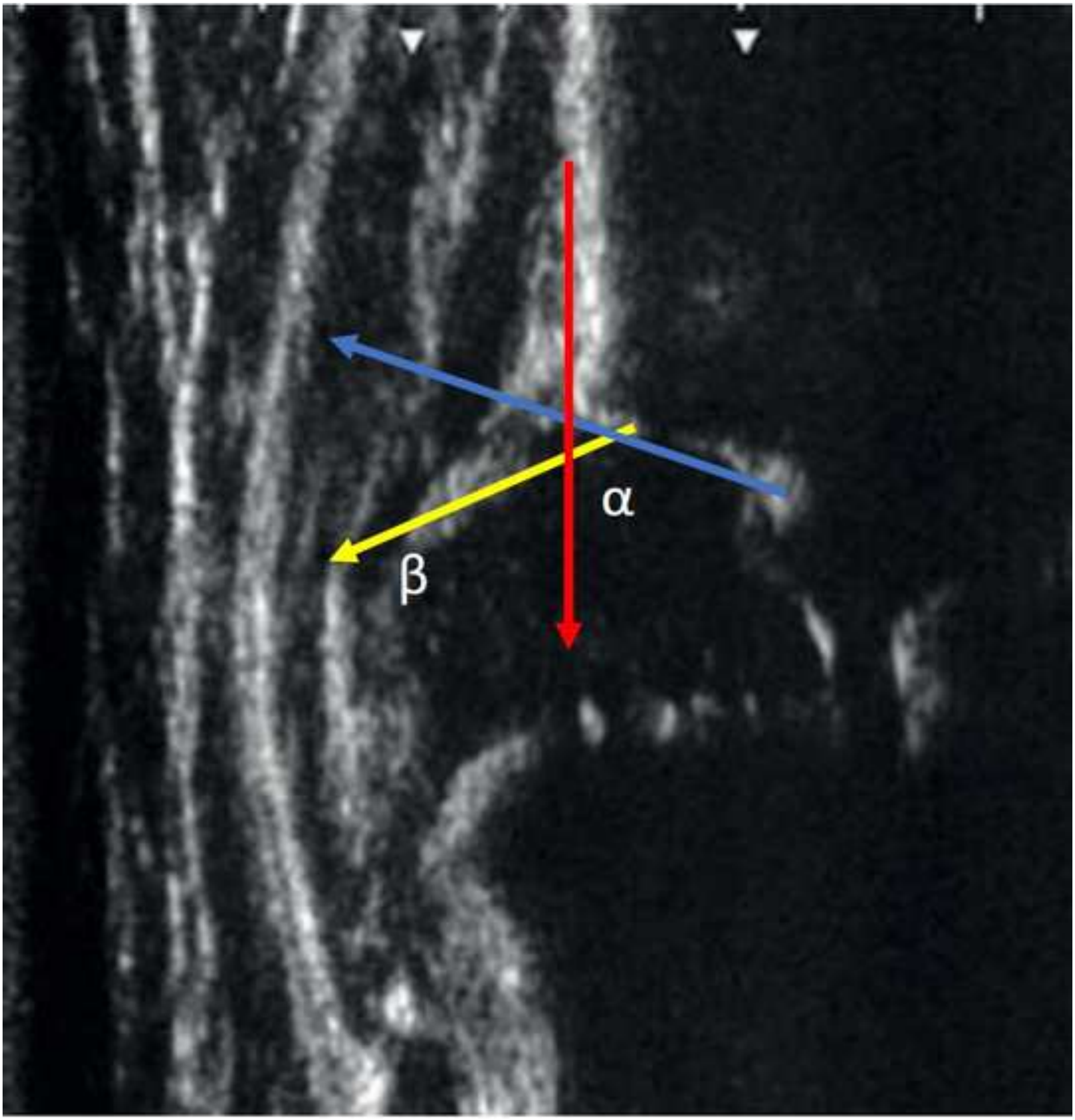

Figure 2

Click here to access/download;Figure;Figure 2.jpg

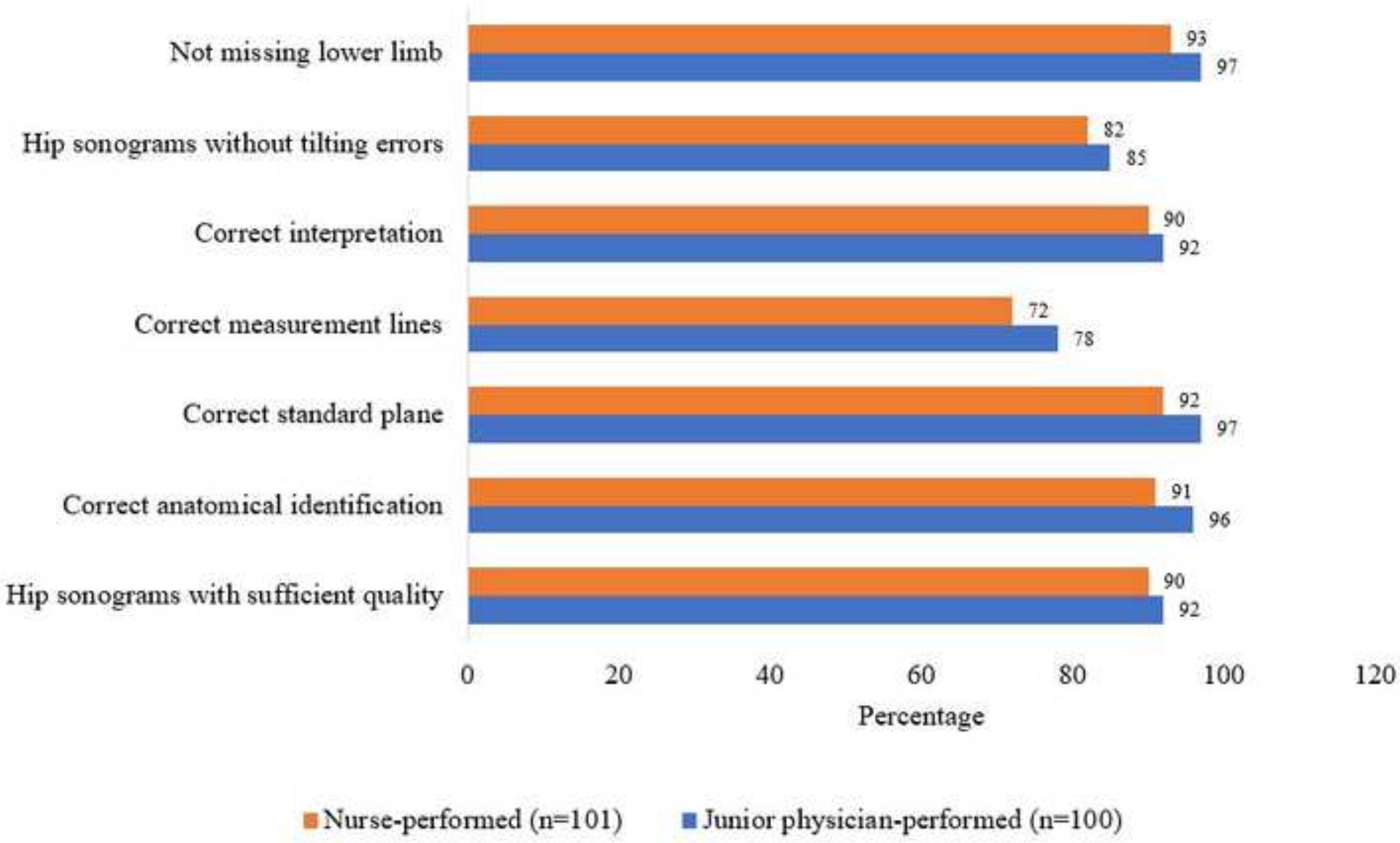

Revised Manuscript with Track Changes

# Comparison of quality and interpretation of newborn~~hip~~ ultrasound screening examinations for developmental dysplasia of the hip by basically trained nurses and ~~to~~ junior physicians with no previous ultrasound experience

*Munkhtulga Ulziibat[1, 2], Bayalag Munkhuu[2], Raoul Schmid[3], Corinne Wyder[4], Thomas Baumann[5], Stefan Essig[5]*

*[1]Department of Health Sciences and Medicine, University of Lucerne, Switzerland*

*[2]National Center for Maternal and Child Health, Ulaanbaatar, Mongolia*

*[3]Baarer Kinderarzt praxis, Baar, Switzerland*

*[4]Kinderärzte KurWerk, Burgdorf, Switzerland*

*[5]Center of Primary and Community Care, Department of Health Sciences and Medicine, University of Lucerne, Switzerland*

**Corresponding author**

Correspondence to: Dr. Munkhtulga Ulziibat

Tel: 976-99175523

Email: umunhtulga@gmail.com

**Formatted:** German (Switzerland)

**Abstract**

**Background:** We are obliged to give babies the chance to profit from a nationwide screening of developmental dysplasia of the hip in very rural areas of Mongolia, where trained physicians are ~~not always available~~ scarce. This study aimed to compare the quality and interpretation of hip ultrasound screening examinations performed by nurses and~~to~~ junior physicians.

**Methods:** A group of 6 nurses and 6 junior physician volunteers with no previous ultrasound experience underwent Graf's standard training in hands-on practice. Newborns were examined before discharge from the hospital, according to the national guideline. Two standard documentation images of each hip were saved digitally. The groups were compared on the proportion of good quality of sonograms and correct interpretation. Two Swiss supervisors' agreed diagnosis according to Graf was considered the final reference for the study purposes.

**Results:** A total of 201 newborns (402 hips or 804 sonograms) were examined in the study, with a mean age of 1.3±0.8 days at examination. Junior physicians examined 100 newborns (200 hips or 400 sonograms), while nurses examined 101 newborns (202 hips or 404 sonograms). The study subjects of the two groups were well balanced for the distribution of baseline characteristics. The study observed no statistically significant difference in the quality of Graf's standard plane images between the providers. Eventually, 92.0% (92) of the physician group and 89.1% (90) of the nurse group were correctly diagnosed as "Group A" (Graf's Type 1 hip) or "Non-Group A" hips (p=0.484).~~ The profession was not significantly associated with incorrect diagnosis in multivariate regression analysis.~~ The most common errors among the groups were a missing lower limb, wrong measurement lines, and technical problems.

**Conclusion:** Our study provides evidence that while there might be a trend of slightly more technical mistakes in the nurse group, the overall diagnosis accuracy is similar to

**Formatted:** English (United States)

**Formatted:** English (United States)

junior physicians after receiving standard training in Graf’s hip ultrasound method.~~,~~ ~~nurses can obtain standard hip sonograms according to Graf with equal quality as junior physicians.~~ However, after basic training, regular quality control is a must and all participants should receive refresher training~~s are needed for nurses and physicians~~. More specifically, nurses need training in the identification of anatomical structures.~~.~~

**Keywords:** hip ultrasound, Graf method, newborn screening, nurse study

## Introduction

Developmental dysplasia of the hip (DDH) is one of the most common disorders of the osteoarticular system with public health priorities in otherwise healthy children. The reported incidence of DDH are quite variable depending on detection methods, ages and diagnostic criteria [1, 2]. Mongolian studies reported a 1-2% incidence of DDH among neonates by ultrasound with Graf's technique [3], an incidence comparable to that in European neonates [4, 5]. DDH is a multifactorial disorder. Contributing factors for the development of DDH are genetic and non-genetic factors [6, 7]. Early diagnosis of developmental dysplasia of the hip (DDH in neonates) is the most critical obligation for achieving the best outcome, using a simple, non-surgical method in the shortest possible time [18-114]. Delayed diagnosis results in losing the potential to remodel the acetabular roof, lengthening the treatment duration, and may cause lifelong disability [125, 136].

The hip ultrasound, according to Graf, which provides basic information about the biomechanical situation, is widely used in several countries as a gold standard of the primary tool in screening for DDH. Unlike other methods, it is standardized, examiner independent, easy to perform, and reproducible. The non-surgical treatment is usually based on the degree of severity of DDH [3, 147]. Graf's method has been shown to be sensitive and specific for the early diagnosis of DDH [15]. Studies have also shown that ultrasonographic screening by Graf is cost-effective [16]. The method cuts through the center of the acetabulum (standard plain) and the hip is evaluated by measuring two angles formed by three lines drawn from three landmarks (lower limb or the bottom of the acetabulum, the acetabular labrum and the lateral edge of the acetabulum). Subsequently, the bony roof angle (alpha angle) and the cartilage roof angle (beta angle) are measured and the hip joint is classified (**Figure 1**).

The introduction of Graf's method of hip ultrasonography and more effective non-surgical treatment in Mongolia since 2010 changed the policy and time of diagnosis and

preventive treatment in children with DDH, resulting in more prevented cases of childhood disabilities [~~8~~4, 5~~9~~]. In 2017, the Mongolian Government approved the hip ultrasound screening as a nationwide screening program. This screening program aims to screen every newborn baby using hip ultrasound by Graf's method and to provide early prevention treatment to eliminate childhood disabilities due to DDH in the country. All major 7 maternity hospitals across Ulaanbaatar, the capital city, and all 21 provincial (aimag) general hospitals are equipped with the necessary equipment and materials; and doctors trained in the method by the Swiss-Mongolian Pediatric Project (SMOPP), a~~the~~ humanitarian aid project. Used Tübinger braces~~orthosis~~ are collected in Switzerland and German-speaking countries and are being reused as a way to help correct the skeletal system.

In Mongolia, a web-based, password-protected platform is used for quality control purpose. The tool enables screeners in all hospitals to upload digital images and annotations exported from the ultrasound machines. Four images (two per hip side; one of them with measured alpha and beta angles according to Graf), a unique identification number, age, sex and diagnosis are required for all examinations. After the upload, quality is controlled by a local expert (a doctor with profound knowledge of the Graf method and at least three years of experience of hip ultrasound and treatment at a provincial hospital). This allowes continuous and reliable reviews of all examinations. In addition, Mongolian supervisors (doctors with profound knowledge of the Graf method and more than 10 years of experience of hip ultrasound and treatment in UB) check examinations from all hospitals and promptly send comments to the experts and screeners.

Since 2019, the screening project has been expanded to major sub-provincial (soum) hospitals responsible for deliveries as a primary source of medical care for nomadic rural residents. However, the sub-provincial hospitals face many significant and unique

**Formatted:** English (United States)

barriers when considering the widespread adoption of newer innovations in health care, such as hip ultrasound screening in newborns. One barrier is a lack of trained physicians capable of performing hip ultrasounds (huge turnover within the doctor's' teams, loss of trained doctors due to high internal and international migrationsdrainage of knowledge and ability, etc.) like in other developing countries [170]. Because of limited resources, physicians are not always available on an around-the-clock or on remote emergency calls from rural areas. Therefore, we feelt obliged to give the babies in the very rural areas the chance to profit from the nationwide screening program. We searched for other possibilities to examine all newborn babies of nomadic families. This study was performed after 3 years of followed bya piloting the expansion project to rural sub-provincial areas. We aimed to compare the quality and interpretation of newborn hip ultrasound examinations (Graf's Type 1 versus "Non-type 1" hips)screening by between basically trained nurses with no previous ultrasound experiences andto trained junior physicians with no previous ultrasound experiences.

## Materials and methods

### *Study design and settings*

A prospective cross-sectional study was conducted in 3 hospitals: a referral hospital in Ulaanbaatar, a provincial (n aimag) general hospital, and a sub-provincial (soum) hospital fromin March to -December 2021. The Ethics Review Committee of the Ministry of OHealth of, Mongolia (MOH), reviewed and approved the study protocol.

### *Study participants*

A sample of 6 nurses and 6 junior physicians wereas self-selected from the nurse and junior physician volunteers in the selected 3 hospitals. The only selection criterion was that they had no previous knowledge or experience in ultrasound. Other criteria were considered obsolete as study participants' volunteering led to a selection of persons that

Formatted: English (United States)

Formatted: English (United States)

would also start ultrasound examinations outside the framework of the study. The six nurses were all certified with bachelor degree (4 years of undergraduate training) from Nursing school of Mongolian National University of Medical Science and had 3-4 years (4 nurses- 3 years and 2 nurses- 4 years) of experience. The six junior physicians were first-year pediatric resident doctors and all received undergraduate training in medicine (six years) from Mongolian National University of Medical Science. All junior physicians had 1-2 years of clinical experience in primary care.

All consecutive newborns born at the 3 hospitals during the study period were ultrasonographically screened by six nurses (two from each hospital) and six junior physicians (two from each hospital). Exclusion criteria were severe or very severe conditions, congenital anomalies, and refusal of parents' written informed consent.

*Preparatory phase*

Nurse- and junior physician- volunteers who consented to participate in the study were trained on using the ultrasound machine through hands-on instruction. Two supervisors with extensive experience in hip sonography and the "ABCD" system facilitated the training [511]. The "ABCD" system is a diagnostic and therapeutic framework based on Graf's technique, SMOPP realized that age-dependent Graf subtypes are irrelevant in the screened neonates in the first week of life. Moreover, it was not practical for the screeners at maternity hospitals to learn all of the different diagnostic subtypes and subsequent treatments outlined by Graf because Graf Type 2c, D and 3 hips are treated in the same way (i.e. flexion-abduction braces). Therefore, the original Graf system required some adjustments into a simpler "ABCD" system. This system is entirely based on the method described by Graf and translates his initial differentiation into four groups: Group A includes Graf Type 1; Group B includes Graf Type 2a; Group C includes Graf Types 2c, D and 3; and Group D includes Graf Type 4 (**Table 1**).

**Formatted:** English (United States)

The nurses and junior physicians ~~who have no prior knowledge or experience in hip sonography~~ received training in basic hip sonography, with emphasis on ultrasound principles, macro, and sono-anatomy of the hip joint, obtaining standard images, and "ABCD" system, measurements, tilting errors, pitfalls and counseling of parents. The standard week courses (15 hours including practical works) were organized in March in sub-provincial, August in provincial, and September 2021 in Ulaanbaatar hospitals. There was no pre-training test since all participants had no previous knowledge or experience in Graf's method of hip ultrasound examinations. However, a post-training test was performed and included questions on theoretical and practical (interpretation and handling) knowledge. The average score of the test was not significantly different between the two groups.

Following the standard introductory course, the nurses and junior physicians completed a week of hip sonography screening and SMOPP's "HipScreen" quality control online platform practice in their hospitals, supervised by the supervisors who provided the training. ~~It was intended that e~~Each nurse and junior physician undertook~~ake around~~ 10 ~~-20~~ newborns' hip sonograms (2 sonograms of right and left hips totaling 80 sonograms), focusing on gaining experience and competency in identifying children who require a referral (non-Group A hips) before testing their skills.

*Data collection*

Newborns were examined on a daily basis before discharge from the hospital ($1^{st}$ or $2^{nd}$ days after birth) according to the national guideline [19~~12~~]. Those who me~~e~~t the selection criteria we~~a~~re recruited in the study, and the following procedures were done:

- counseling of mothers and brief information about DDH
- hip ultrasound examination

- information about the next steps according to hip ultrasound examination results.

All eligible newborns were examined using a GE LOGIQ series (C3) ultrasound machine with a 7 to 8-MHz linear-array transducer. The examinations were performed and interpreted according to the national guideline based on the SMOPP's "ABCD" system [1~~2~~8], the modified Graf's system by the trained nurses or junior physicians depending on the days (odd days for nurses and even days for the junior physicians). Two standard documentation images of each hip were saved digitally using the HipScreen system. Follow-up control and management followed ~~up~~ the National guideline [19~~3~~].

Two Swiss supervisors checked all the nurses' and junior physicians' hip ultrasound images. The two supervisors were blinded to the nurses and doctor~~examiner~~s. All supervisors are internationally certified and have at least 10 years of experience in Graf's method of hip ultrasonography. Their final agreed diagnosis was considered the final reference for the study purposes. All supervisors interpreted all examinations (nurses and junior physicians); if discordant, a discussion led to a consensual agreement~~mutual result~~.

***Outcome measurement***

1. The following 4 indicators measured the quality of the newborn hip ultrasound images:

a) Anatomical identification is "correct" if all anatomical structures, according to Graf [3, 12] are identified in the hip sonogram. If at least~~only~~ one structure is missing, the sonogram is considered "incorrect".

b) The standard plane is "correct" if all 3 points according to Graf's criteria [3, 12] (Lower limb of the bony ilium, Mid-portion of the acetabular roof, and Acetabular

labrum) are visible in the hip sonogram. If at least~~only~~ one structure is missing, the sonogram is considered “incorrect”.

c) Three lines (baseline, bony, and cartilaginous roof lines) are measured “correct” or “incorrect” according to Graf’s criteria [3, 12].

d) The tilting was measured as “yes” or “~~/~~no” depending on the absence/presence of any tilting errors according to Graf’s criteria [3, 12]. In order to get a standard plain sonogram the transducer should be placed vertically on the hip joint. Tilted positions of the transducer can lead to significant examinations errors (usually a mechanical transducer guide prevents tilting errors by reducing the degrees of variability).

2. The correct interpretation: The screeners (nurses and junior physicians) were requested to comment on the “Group A” (Graf’s Type 1 hip) or “Non-Group A” (Graf’s non-Type 1 hip). Then it was checked against the reference interpretation performed by the Swiss experts. The interpretation was defined as “correct” or “incorrect”.

**Statistical analyses**

Statistical analyses were performed using Stata 16 (Stata Corporation) software. Continuous variables were summarized as mean, standard deviation, and non-continuous variables as frequencies and percentages. A comparison of two groups (nurses and junior physicians) was performed using the $\chi 2$ test, Fisher’s exact test, and Student’s t-tests depending on the variables, with a significance level of 0.05 or less.

Univariate comparison of correct and incorrect diagnosis was performed using Fisher’s exact test. ~~and the variables with a significance level of 0.2 or less were initially included in a logistic regression model. Those with highest likelihood ratio p-value were removed one-by-one based on backward elimination strategy until the model contained only significant variables with p ≤ 0.05.~~ In the main analysis, we used the child and not the hip sonogram (2 sonograms per hip or 4 hip sonograms per child) as the unit of analysis.

**Formatted:** English (United States)

**Formatted:** English (United States)

**Formatted:** English (United States)

**Formatted:** English (United States)

If a child had hip sonograms with different qualities, we evaluated the child based on the worst quality.

Power calculations indicate that we need to include at least 92 subjects in each group based on the assumptions that 1) the expected proportion of insufficient quality of hip ultrasound of the junior physician group is 10%, 2) the expected proportion of insufficient quality of hip ultrasound of the nurse group is 25%, 3) the alpha level is 0.05, and 4) the required power is 80%.

**Formatted:** English (United States)

## Results

A group of 6 nurses and 6 junior physician volunteers with no previous ultrasound experience underwent Graf's standard training in hands-on practice and didactic instruction. The mean age of the junior physicians and nurses was 25±1.4 and 26.3±0.8 years, respectively. All were female.

A total of 201 newborns (402 hips or 804 hip sonograms) were examined in the study, with a male/female ratio of 118/83 and a mean age of 1.3±0.9 days at examination. Junior physicians examined 100 newborns (200 hips or 400 hip sonograms), while nurses examined 101 newborns (202 hips or 404 hip sonograms). The study subjects/newborns were well balanced for the distribution of baseline characteristics, such as mean age, sex, birth weight, and hospitals. (**Table 2**)

As shown in Figure 2~~**Table 2**~~, of 100 newborns examined in the junior physician-performed group and 101 newborns examined in the nurse-performed group, the proportion of sufficient quality of hip sonograms was 92.0% and 89.1% (p=0.484), respectively. In the anatomical identification, 96 (96.0%) and 91 (90.1%) newborns' hip sonograms in the respective groups were correct (p=0.100). The incorrect standard plane was recorded in comparatively few proportions in both groups (3.0% in the junior physician's group vs. 8.9% in the nurse's group; p=0.077).

The proportion of measurement lines was 78.0% in the junior physician-performed group, while in the nurse-performed group, the proportion was 71.3% (p=0.274).

Eventually, 92.0% (90) of the junior group and 89.1% (90) of the nurse group (p=0.484) correctly diagnosed the "Group A" (Graf's Type 1 hip) or "Non-Group A" (Graf's non-Type 1 hip). (Figure 2)

A common error among the groups was the tilting (17.0% in the junior group vs. 16.8% in the nurse's group, p=0.975), followed by the missing the lower limb (3.0% in the junior group vs. 7.9% in the nurse's group, p=0.125). (Figure 2~~**Table 3**~~)

Univariate comparisons of demographic and hip ultrasound (Graf method) examination characteristics of "correct" and "incorrect" diagnosis are displayed in **Table 3**. The incorrect diagnosis was significantly more likely to be due to smaller birth weight of newborns (p=0.012), incorrect anatomical identification (p<0.0001), incorrect measurement lines (p<0.0001), missing lower limb and tilting errors (p<0.002).

~~**Table 5** summarizes the independent variables for being incorrectly diagnosed using multivariate logistic regression analysis. The odds of incorrect diagnosis status was increased significantly in children having small birth weight (smaller for each 100 grmas; OR=0.01, 95% CI 0.01, 05) and having incorrect anatomical identification (OR=1.1, 95% CI 0.1, 0.5) and incorrect lines (OR=20.4, 95% CI 5.2, 79.6), and missing lower limb (OR=2.2, 95% CI 1.3, 3.2). Nurse performed hip examinations were likely to be incorrect (OR=1.3, 95% CI 0.4, 4.4), but it was not statistically significant (p=0.595).~~

**Discussion**

The study provides evidence that nurses could be a practical alternative where suitable hip ultrasound-trained doctors are unavailable. The "Graf's standard image" quality success proportion of 92.0% and 89.1% were observed for junior physicians' and

**Formatted:** English (United States)

nurses' groups, respectively. Furthermore, the study revealed similar "Group A" or "Non-Group A" hips' correct identification rates in both groups. ~~The profession was not significantly associated with incorrect diagnosis in multivariate regression analysis.~~The most common error among the groups was missing the lower limb.

In developing countries, implementing and sustaining any newborn screening, including newborn hip ultrasound, is challenging due to potential political instability, lack of trained human resources and less developed public health systems, etc [20~~13~~]. Over the past decade, the humanitarian Swiss-Mongolian Pediatric Project SMOPP, commissioned by the MOH in Mongolia, has established a newborn ultrasound screening and early conservative treatment program for developmental dysplasia of the hip [4, 5~~9~~]. The obligation to implement a DDH ultrasound screening program, especially in a country with limited resources, requires plausible, simple structures and straightforward algorithms. These requirements led to two paradigms in the implementation of the SMOPP: a) application of the Graf's ultrasound method [3, 12] as early as possible in the newborn period in a simplified form with only 4 intervention groups [18~~11~~], and b) uniform, simple, cost-effective outpatient treatment of all DDH severity levels with a flexion-abduction device performed by the parents under pediatric surveillance [3, 12, 18~~11~~].

In Mongolia, the Graf's method is used in a simplified modified ABCD adaptation [18~~11~~], which combines the Graf types into intervention groups according to therapeutic aspects (A = normal; B = physiologically immature, worthy of control; C = DDH, in need of immediate therapy through flexion-abduction-braces~~orthosis~~; D = dislocated). Knowing the high maturation potential in the first 3 months of life [21~~4~~], the strategy must be implemented as early as possible after birth. The feasibility of the concept in Mongolia was proven by a cohort study [4]~~8~~ and subsequent statistical analyses [5~~9~~]. All cases (n=107) of DDH discovered in newborns were cured using the Tübingen

**Formatted:** English (United States)

bracessplint [2215]. In addition, the hip ultrasound screening has been well accepted by local newborn hip ultrasound screeners (e.g., neonatologists and pediatricians) even though the screening was comparatively new to the providers. Nevertheless, the screening implementation has not been smooth, especially in prominent sub-provincial hospitals because physicians are not always available when they are on remote emergency calls from remote rural areas.

The need for a possible alternative approach to screen every newborn baby for DDH by ultrasound is obvious when trained doctors are not available, especially in rural areas. To address the challenge of detecting "non-Group A" (Graf's non-Type 1 hip) cases before discharge from maternity hospital (usually, after birth, newborns stay 1-2 days), it might be wise to train nurses since nurses are a more stable group in terms of mobility or changing workplace in the country [2316]. This is especially crucial in rural hospitals where nomadic residents receive maternity and health services. Thus our study aimed to evaluate the feasibility of nurses without prior experience in ultrasonography to obtain the standard plane images of newborn hips. The study observed no statistically significant difference in the quality of Graf's standard plane images between the providers: junior physicians and nurses. Nevertheless, the study suggests that the nurses might need more training to correctly identify anatomical structures. Also in our study the profession was not significantly associated with incorrect diagnosis in multivariate regression analysis.

Although the role of nurse-led hip ultrasonography is insufficiently studied, Professor Graf, the founder of the hip ultrasound method, repeatedly mentioned that the nurse-performed hip ultrasound has good accuracy. There is a study that aimed to evaluate healthcare workers' ability to classify ultrasound images into a Graf system [2417]. The study team consisted of 3 physicians and 4 nurses at the infant health care system, with no previous ultrasound experience in the Netherlands. After theoretical and practical

**Formatted:** English (United States)

training, seven nurses and physicians of the participating infant health centers reported their findings on hip sonograms of 80 children. This was repeated five months later. From the two evaluation moments, the intra-observer agreement and the inter-observer agreement were determined. Based on the study results, the authors concluded that the inter- and intra-observer agreement is comparable to similar studies in which the participants had a professional background in ultrasound examination. The level of agreement of the trainees in the perspective of the screening process was considered sufficient [24~~17~~]. However, this study addressed only the intra- and inter-observer variability in reading a sonogram. Therefore, the findings of this study and our results cannot be compared.

On the other hand, nurses are key players in the newborn screening program, and their role is crucial in increasing newborn screening coverage [25~~18~~] Considered front liners, nurses are usually the first contact with parents in a primary care setting or health facility, allowing them to advocate and educate parents on newborn screening. Besides these advantages recently, several studies have shown that appropriately trained nurses can perform some ultrasound scans [26~~19~~-31~~24~~]. Although the role of nurse-led hip ultrasonography is poorly studied, it has been described as helpful in the placement of central [26]~~21~~ and peripheral intravenous lines [26, 28~~19~~] in patients with difficult access, for the focused assessment in emergency situations [30, 32~~3~~, 33~~25-26~~] and patients with urologic [29~~22~~, 34~~27~~], obstetric [31~~22~~, 35~~28~~], and cardiac diseases [2~~0~~7].

In the present study, common errors were missing the lower limb in both groups and subsequent measurement bias that led to the wrong diagnosis. It is mandatory to check the usefulness of a hip sonogram according to the Graf method: a) to identify the lower limb of the os ilium, b) the precise middle plain of the acetabulum roof, and c) the labrum (lower limb – plain - labrum); and errors of tilting must be excluded. Such errors may eventually lead to incorrect procedures that may cause unnecessary over or under-

treatment for babies without a quality control system [36, 37~~29,30~~]. However, in the study, a quality control system allowed reliable review of the diagnosis and treatment of DDH and continuous education of nurses and junior doctors.

The current study has some potential limitations. 1) The generalizability of this study into the whole nurses’ community of different levels of care might be limited because only six nurses and six physicians performed the examinations. However, we tried to have a delegation from different levels of care hospitals, e.g., from referral, provincial and sub-provincial hospitals. Concerning the study’s limitations, the nurses’ or junior physicians’ characteristics might result in variations of skills related to image acquisition and interpretation which could affect the quality of the hip ultrasound scan. 2) The nurses’ and junior physicians’ evaluations of newborns’ hips were not validated against a reference method such as computerized~~d~~ tomography or magnetic resonance imaging. However, Graf’s method of hip ultrasound is standardized, it accepts only a standard plane cut of the acetabulum, and hence it is also reproducible. Our study focused on evaluating whether nurses and junior physicians can obtain the correct plane of the hip sonograms and identify “non-Group A” or “Graf’s non-Type 1” hips~~)~~. The study did not address the intra- and inter-observer agreement regarding hip sonograms. Nevertheless, the quality and interpretation of the nurse- and junior physician-performed hip ultrasound images were compared.

**Conclusion**

Our study provides evidence that while there might be a trend of slightly more technical mistakes in the nurse group, the overall diagnostic accuracy is similar ~~nurses can obtain standard hip sonograms with equal quality as~~ to junior physicians after receiving standard training in Graf’s hip ultrasound method. However, after a basic standard training, regular quality control is a must and all participants should receive~~and~~ refresher

trainings. ~~are compulsory for both nurses and physicians.~~More specifically, nurses need training in the identification of anatomical structures.


## Acknowledgements

We would like to thank all families, nurses and physicians who participated in this study. We would also like to thank our colleagues who provided expert advice and are members of the Swiss Mongolian Pediatric project and the Mongolian Society of Developmental Dysplasia of the Hip Prevention.


**Formatted:** English (United States)

**References**


1. Loder RT, Skopelja EN. The epidemiology and demographics of hip dysplasia. ISRN Orthop. 2011; 2011:238607. https://doi.org/10.5402/2011/ 238607

2. Shipman SA, Helfand M, Moyer VA, Yawn BP. Screening for developmental dysplasia of the hip: a systematic literature review for the US preventive services task force. Pediatrics. 2006; 117(3):557–576. https://doi.org/10. 1542/peds

3. Graf, R. Fundamentals of sonographic diagnosis of infant hip dysplasia. J. Pediatr. Orthop. 1984, *4*, 735–740.

4. Munkhuu B, Essig S, Renchinnyam E, Schmid R, Wilhelm C, et al. Incidence and treatment of developmental hip dysplasia in Mongolia: a prospective cohort study. PLoS One. 2013; 8(10):e79427. https://doi.org/10.1371/journ al.pone.0079427

5. Ulziibat M, Munkhuu B, Schmid R, Baumann T, Essig S. Implementation of a nationwide universal ultrasound screening programme for developmental dysplasia of the neonatal hip in Mongolia. J Child Orthop. 2020; 14. https://doi.org/10.1302/1863-2548.14.200029.

6. Carter C, Wilkinson J. Genetic and environmental factors in the etiology of congenital dislocation of the hip. Clin Orthop Relat Res. 1964;33: 119–128.

7. Ulziibat M, Munkhuu B, Bataa AE, Schmid R, Baumann T, Essig S. Traditional Mongolian swaddling and developmental dysplasia of the hip: a randomized controlled trial. BMC Pediatr. 2021 Oct 13;21(1):450. doi: 10.1186/s12887-021-02910-x. PMID: 34641800; PMCID: PMC8513275.

8. 1.Joseph Gerard O'Beirne, Konstantinos Chlapoutakis, Sattar Alshryda, et al. international consensus meeting on evaluation of developmental dysplasia of the hip. Ultraschall in Med 2019; 40:454-464.


**Formatted:** German (Switzerland)

**Formatted:** German (Switzerland)

9. Thallinger C, Pospischill R, Ganger R, et al. Long term results of a nationwide general ultrasound screening system for developmental disorders of the hip: the Austrian hip screening program. J Child Orthop. 2014; 8:3–10. doi: 10.1007/s11832-014-0555-6.

10. Graf R, Baumgartner F, Lercher K, Scott S, Benaroya A. Hip Sonography. Diagnosis and Management of Infant Hip Dysplasia. 2006. Springer ISBN 3-540-30957-8

11. Kotlarsky P, Haber R, Bialik V, Eidelman M. Developmental dysplasia of the hip: What has changed in the last 20 years? World J Orthop. 2015; 6(11):886-901. doi: 10.5312/wjo.v6.i11.886. PMID: 26716085; PMCID: PMC4686436.

12. Brdar R, Petronic I, Nikolic D, Lukac M, Cirovic D, Blagojevic T. Walking quality after surgical treatment of developmental dysplasia of the hip in children. Acta Orthop Belg 2013; 79(1):60-63.

13. Fujii M, Nakashima Y, Jingushi S, et al. Intraarticular findings in symptomatic developmental dysplasia of the hip. J Pediatr Orthop 2009; 29: 9–13.

14. Graf R. The diagnosis of congenital hip-joint dislocation by the ultrasonic compound treatment. Arch Orthop Trauma Surg. 1980; 97:117–133. doi:10.1007/BF00450934

15. O'Beirne JG, Chlapoutakis K, Alshryda S, et al. International interdisciplinary consensus meeting on evaluation of developmental dysplasia of the hip. Ultraschall Med 2019; 40:454-464

16. Thaler M, Biedermann R, Lair J, Krismer M, Landauer F. Cost-effectiveness of universal ultrasound screening compared with clinical examination alone in the diagnosis and treatment of neonatal hip dysplasia in Austria. J Bone Joint Surg [Br] 2011; 93-B:1126-1130

17. World Health Organization. Regional Office for Europe, European Observatory on Health Systems and Policies, Bolormaa, T, Natsagdorj, Ts, Tumurbat, B. et al.

**Formatted:** English (United States)

**Formatted:** English (United States)

Mongolia: health system review. World Health Organization. Regional Office for Europe. 2007. https://apps.who.int/iris/handle/10665/107845

18. Baumann T, Schmid R, Essig S, et al ABCD OF DDH. Zumsteg Druck AG. 2017 https://www.thieme-connect.com/products/ejournals/html/10.1055/s-0043-110690.

19. Ministry of Health Mongolia. National guideline of newborn ultrasound screening and management for the developmental dysplasia of the hip. Ministry of Health Mongolia. Ulaanbaatar. 2021

20. Therrell BL,Padilla CD. Barriers to implementing sustainable national newborn screening in developing health systems. International Journal of Pediatrics and Adolescent Medicine. 2014; 1(2):49-60.

21. Tschauner C, Klapsch W, Baumgarthner A, Graf R. Maturation curve of the ultrasonographic alpha angle according to Graf's untreated hip joint in the first year of life. Z Orthop Ihre Grenzgeb 1994; 132:502-504.

22. Baumann T, Munkhuu B, Chuluunbaatar B, Schmid R. 4-year follow-up of ultrasound-based diagnosis and non-surgical treatment of developmental dysplasia of the hip in Mongolia: a prospective cohort study. Ultraschall in der Medizin. 2016; 37(S 01) DOI: 10.1055/s-0036-1587724. https://www.thieme-connect.com/products/ejournals/abstract/10.1055/ s-0036-1587724

23. World Health Organization. Regional Office for Europe, European Observatory on Health Systems and Policies, Bolormaa, T, Natsagdorj, Ts, Tumurbat, B. et al. Mongolia: health system review. World Health Organization. Regional Office for Europe. 2007. https://apps.who.int/iris/handle/10665/107845

24. Ramwadhdoebe S, Sakkers RJ, Uiterwaal CS, Boere-Boonekamp MM, Beek FJ. Evaluation of a training program for general ultrasound screening for developmental

**Formatted:** English (United States)

**Formatted:** German (Switzerland)

**Formatted:** German (Switzerland)

**Formatted:** English (United States)

dysplasia of the hip in preventive child health care. Pediatr Radiol. 2010; 40(10):1634-9. doi: 10.1007/s00247-010-1689-4. Epub 2010 Jun 12. PMID: 20544189.

25. DeLuca J, Zanni KL, Bonhomme N, Kemper AR. Implications of newborn screening for nurses. J Nurs Scholarsh. 2013; 45(1):25-33. doi: 10.1111/jnu.12005. Epub 2013 Jan 31. PMID: 23368429.

26. Stokowski G, Steele D, Wilson D. The use of ultrasound to improve practice and reduce complication rates in peripherally inserted central catheter insertions: final report of investigation. J Infus Nurs 2009; 32:145–55.

27. Colquhoun SM, Carapetis JR, Kado JH, Reeves BM, Remenyi B, May W, Wilson NJ, Steer AC. Pilot study of nurse-led rheumatic heart disease echocardiography screening in Fiji - a novel approach in a resource-poor setting. Cardiol Young 2013; 23:546–52.

28. Weiner SG, Sarff AR, Esener DE, Shroff SD, Budhram GR, Switkowski KM, Mostofi MB, Barus RW, Coute RA, Darvish AH. Single-operator ultrasound-guided intravenous line placement by Emergency Nurses reduces the need for physician intervention in patients with difficult-to-establish intravenous access. J Emerg Med 2013; 44: 653–60.

29. Paez A, Ju_arez N, Mar_ın M, Del Vigo S, Redondo E, Dur_an M. Concordance analysis of ultrasonographic diagnoses by urology nurses. Arch Esp Urol 2012; 65: 872–8.

30. Bowra J, Forrest-Horder S, Caldwell E, Cox M, D'Amours SK. Validation of nurse-performed FAST ultrasound. Injury 2010; 41: 484–7.

31. Menihan CA. Limited obstetric ultrasound in nursing practice. J Obstet Gynecol Neonatal Nurs. 2000; 29(3):325-30. doi: 10.1111/j.1552-6909.2000.tb02054.x. PMID: 10839581.

**Formatted:** English (United States)

**Formatted:** English (United States)

32. Bloom BA, Gibbons RC. Focused Assessment with Sonography for Trauma. In: StatPearls [Internet]. Treasure Island (FL): Stat Pearls Publishing; 2022. https://www.ncbi.nlm.nih.gov/books/NBK470479/

33. Wayne Varndell, Michelle Topacio, Carmel Hagness, Hayley Lemon, Deborah Tracy. Nurse-performed focused ultrasound in the emergency department: A systematic review. Literature review 2018; 21(4):121-130. DOI:https://doi.org/10.1016/j.auec.2018.09.30.

34. Nandwana SB, Walls DG, Ibraheem O, Murphy F, Tridandapani S, Cox K. Image-Guided Nontargeted Renal Biopsies Performed by Radiology-Trained Nurse Practitioners: A Safe Practice Model. J Am Coll Radiol. 2016; 13(7):819-21.

35. Marilyn Stringer, Susan R. Miesnik, Linda P. Brown, Lynne Menei, George A. Macones. Limited Obstetric Ultrasound Examinations: Competency and Cost. Clinical research 2003; 32:307-312. DOI:https://doi.org/10.1177/0884217503253452.

36. Tschauner, C, Matthissen, H. Hip Sonography with Graf-method in Newborns: Checklists help to avoid mistakes. OUB 2012; 1:7–8.

37. Graf R, Mohajer M, Plattner F. Hip sonography update. Quality management, catastrophes - tips and tricks. Med Ultrason 2013; 15:299-303.

**Formatted:** English (United States)

**Formatted:** English (United States)

**Figure 1. A hip sonogram.**

A. Three landmarks of the standard plain.- 1. Standard cut (red arrow), 2. Lower limb or the bottom of the acetabulum (yellow circle), 3. Acetabular labrum and the lateral edge of the acetabulum (blue circle).

B. Hip is evaluated by measuring two angles formed by three lines drawn from three landmarks. 1. Basic line (red), 2. Bony roof line (blue), 3. Cartilaginous roof line (yellow)

**Formatted:** English (United States)

**Figure 2. Hip ultrasound examination performance of the nurses’s and junior physicians‘ groups**

Formatted: English (United States)

**Table 1. Comparison of Graf types and ABCD groups for hip ultrasound**

* Group B hips were defined as angle alpha minus the age in weeks = between 50°-60°

| **Graf type** | **Graf angle $\alpha$ (°)** | **Graf angle $\beta$ (°)** | **Graf therapy** | **ABCD group** | **ABCD angle $\alpha$ (°)** | **ABCD therapy** |
|---|---|---|---|---|---|---|
| 1a | > 60 | < 55 | None | A | > 60 | None |
| 1b | | > 55 | None | | | |
| 2a | 50–59 | | Control | B | > 50 ≤ 60* | Control |
| 2a+ | | | Control | | | |
| 2a- | | | Spreading device/Pavlik | C | < 50 | Tübingen braces |
| 2b | | | | | | |
| 2c stable | 43–49 | < 77 | | | | |
| 2c unstable | | | Plaster | | | |
| D | | > 77 | | | | |
| 3a | < 43 | | Extension | | | |
| 3b | | | | | | |
| 4 | | | Operation | D | - | Tübingen braces/Operation |

**Formatted:** English (United States)

**Formatted:** Justified

**Formatted:** English (United States)

**Table 2. Newborn characteristics**

| **Characteristics** | **All N=201 n(%)** | **Junior physician-performed N=100 n(%)** | **Nurse-performed N=101 n(%)** | **P value** |
|---|---|---|---|---|
| Mean age at hip ultrasound examination, days (mean±sd) | 1.3±0.9 | 1.3±0.8 | 1.3±0.9 | 0.2877***** |
| **Sex** | | | | 0.711** |
| Male | 118 (58.7) | 60 (60.0) | 58 (57.4) | |
| Female | 83 (41.3) | 40 (40.0) | 43 (42.6) | |
| Hospital | | | | 0.078** |
| Referral | 98 (48.8) | 55 (55.0) | 43 (42.6) | |
| Provincial and sub-provincial | 103 (51.2) | 45 (45.0) | 58 (57.4) | |
| Mean birth weight, kg (mean±sd) | 3.2±0.2 | 3.2±0.2 | 3.2±0.2 | 0.820***** |

**t-test, **Chi$^2$ test*

~~**Table 2. The quality of the newborn hip ultrasound images**~~

| ~~**Characteristics**~~ | ~~**Junior physician-performed N=100**~~ | ~~**Nurse-performed**~~ | ~~**P value***~~ |
|---|---|---|---|

| | n(%) | N=101 n(%) | |
|---|---|---|---|
| Quality of hip sonograms | | | 0.484 |
| – Sufficient | 92 (92.0) | 90 (89.1) | |
| – Insufficient | 8 (8.0) | 11 (10.9) | |
| Anatomical identification | | 0.245 | 0.100 |
| – Correct | 96 (96.0) | 91 (90.1) | |
| – Incorrect | 4 (4.0) | 10 (9.9) | |
| Standard plane | | | 0.077 |
| – Correct | 97 (97.0) | 92 (91.1) | |
| – Incorrect | 3 (3.0) | 9 (8.9) | |
| Measurement lines | | | 0.274 |
| – Correct | 78 (78.0) | 72 (71.3) | |
| – Incorrect | 22 (22.0) | 29 (28.7) | |
| Diagnosis | | | 0.484 |
| – Correct | 92 (92.0) | 90 (89.1) | |
| – Incorrect | 8 (8.0) | 11 (10.9) | |

**Chi² test*

**Table 3. Common errors of the study groups**

| ~~Errors~~ | ~~Junior physician-performed N=100 n(%)~~ | ~~Nurse-performed N=101 n(%)~~ | ~~P value*~~ |
|---|---|---|---|
| ~~Tilting~~ | | | ~~0.975~~ |
| ~~Yes~~ | ~~15 (15.0)~~ | ~~19 (18.8)~~ | |
| ~~No~~ | ~~85 (85.0)~~ | ~~82 (81.2)~~ | |
| ~~Missing lower limb~~ | | | ~~0.125~~ |
| ~~Yes~~ | ~~3 (3.0)~~ | ~~8 (7.9)~~ | |
| ~~No~~ | ~~97 (97.0)~~ | ~~93 (92.1)~~ | |

~~*Fisher exact~~

**Table ~~3~~4. Univaraite analysis of ~~D~~demographic and hip ultrasound examination characteristics of correct and incorrect diagnosis**

Formatted: English (United States)

| **Characteristics** | **Correct diagnosis N=182** | **Incorrect diagnosis N=9** | **p-value** |
|---|---|---|---|
| Age of newborns (CI) | 1.3 ± 0.9 (1.2 - 1.5) | 1.2 ± 0.7 (0.7 - 1.5) | 0.439 |
| Mean birth weight (CI) | 3.2 ±0.2 (3.2 - 3.3) | 3.1 ± 0.2 (2.9 - 3.2) | 0.0123 |
| Hospital | | | 0.275 |
| UB | 91 (50.0) | 7 (36.8) | |
| rural | 91(50.0) | 12 (63.2) | |

| | | | |
|---|---|---|---|
| Performed by | | | 0.484 |
| junior physician | 92 (50.6) | 8 (42.1) | |
| nurse | 90 (49.4) | 11 (57.9) | |
| Anatomical identification | | | <0.0001 |
| correct | 182 (100.0) | 5 (26.3) | |
| incorrect | 0 | 14 (73.7) | |
| Standard plain cut | | | <0.0001 |
| correct | 182 (100.0) | 7 (36.8) | |
| incorrect | 0 | 12 (63.2) | |
| Measurement lines | | | <0.0001 |
| correct | 148 (81.3) | 2 (10.5) | |
| incorrect | 34 (18.7) | 17 (89.5) | |
| Tilting errors | | | 0.002 |
| yes | 26 (14.3) | 8 (42.1) | |
| no | 156 (85.7) | 11 (57.9) | |
| Lower limb | | | <0.0001 |
| yes | 0 | 11 (57.9) | |
| no | 182 | 8 (42.1) | |

***Fisher exact***

**Table 5. Significant factors related to incorrect diagnosis from final logistic regression model**

| Characteristics | Odds ratio | 95% confidence interval | p value |
|---|---|---|---|
| Birth weight of newborn () | 0.01 | 0.01-0.5 | 0.021 |
| Incorrect anatomical identification | 1.1 | 0.1-0.5 | <0.001 |
| Incorrect lines | 20.4 | 5.2-79.6 | <0.001 |
| No lower limb | 2.2 | 1.3-3.2 | <0.001 |
| Nurse performed | 1.3 | 0.4-4.4 | 0.595 |

**Editor Comments**

The manuscript has been evaluated by five reviewers, and their comments are available below. The reviewers have raised a number of concerns throughout your manuscript that need attention. They recommend additional background information in the Introduction to better introduce the topic. They also feel the Methods section may benefit from further clarification, particularly as it pertains to the education of the trainees, and the inclusion of statistical power calculations which may validate conclusions drawn from your study. It is also suggested to include additional images which may enhance presentation for the reader. Could you please revise the manuscript to carefully address the concerns raised?

**Response:** Thank you and the reviewers very much for the opportunity to submit a revision of our manuscript. We added additional background information, information on the education of the trainees and statistical power calculations, as well as images. Below, we respond to each point raised by the reviewers. If we can provide any additional information or make any other changes, please do not hesitate to let us know.

The manuscript has important value for dissemination, given the needs at the primary care level in many countries. The study and results are well designed and explained. However, perhaps from the language and writing parts there are several unclear expressions that may confuse the readers. Thus, I have made several suggestions so that you and authors can review, revise, correct and then re-submit. Thank you.

**Response:** Thank you for your support. We are not sure which suggestions came from you as the comments of you and the reviewers were submitted in three parts (text in the e-mail, PDF and Word file). We respond to each point from all three parts below and carefully address the concerns raised.

**Reviewer 1**

There are several issues in this manuscript. Please see the attached PDF sent with the review, where I place several suggestions to improve the data analysis and manuscript writing, for better understanding and integrity.

**Response:** Thank you for your comments. They were very helpful in crafting our revision.

Line 2: It seems better to change the word to “and” instead of “to” (to avoid confusion in readers)

**Response:** We have modified the title of the manuscript **(lines: 1-3)**

Line48: It would be good to start with some form of "prevalence" of dysplasia and other developmental problems in the country and region, to put into context what you will be developing...

**Response:** The information was added (**lines: 50-56**)

Line48: Add something like "...in the neonates" to make it clear to whom it is done.

**Response:** It was added (**line: 57**)

Line 52: "the Graf method" (it would be good to add one sentence briefly explaining what it is, for the benefit of readers who are not very familiar with it).

**Response:** Information and picture were added (**lines: 66-73 and Figure 1**)

Line 65: insert "the "

**Response:** It was corrected (**line: 82**)

Line 66: The Swiss- Mongolian Pediatric Project

**Response:** Thank you. It was corrected (**line: 83**)

Line 67: a humanitarian

**Response:** It was corrected (**line: 83**)

Line 67: Add braces to make it more clear

**Response:** It was changed. (**line: 84**)

Line 68: Add "as a way to help correct the skeletal system" (to make the phrase clearer)

**Response:** The phrase was added (**lines: 85-86**)

Line 79: add "program"

**Response:** It was added (**line: 109**)

Line80: This is not clear. Did you mean to say "This study will be followed..." (because until now, there is no mention of a "study" so this surprises the reader). Please correct the phrase...

**Response:** The sentences were modified (**lines: 109-115**)

Line82: "and by"

**Response:** It was corrected (**line: 114**)

Line 88: from March to December 2021",

**Response:** It was corrected (**line: 120**)

Line89: "the Mongolia Ministry of Health (MOH)"

**Response:** It was corrected (**lines:120-121**)

Line 92: "self-selected" means they volunteered? Also, were there any criteria for such selection (e.g., more experienced, working at least one year, etc.?). It is important to give the reader as much information about this selection as possible.

**Response:** We agree that the term “self-selected” was unclear. We adapted the paragraph (**lines: 124-127**)

Line 100: Again, explain a little bit about this ABCD system” for the benefit readers…

**Response:** The explanation was added (**lines: 142-151, Table 1**)

Line 101: "The selected nurses and junior physicians, who had no prior knowledge or experience in hip sonography" (to make it clearer and accurate).

**Response:** We removed the sentence and now mention the inclusion criterion under “Study participants”. (**lines: 124-127**)

Line110: This is NOT clear. "It was intended" means it was DONE or was put as an objective? Does it mean that they at least undertook 10 sonograms to gain experience? This has to be clear, otherwise the reader doesn't know what happened...

**Response:** Thank you, it was clarified (**lines:165-168**)

Line116: "met" (past tense)

**Response:** It was corrected (**line: 171**)

Line117: "were" (past tense)

**Response:** It was corrected (**line: 172**)

Line123: add “ultrasound machine”

**Response:** It was added (**lines:177-178**)

Line 124: It is not absolutely necessary, but you could add a picture/graph to make it clearer - ?

**Response:** Thank you for the suggestion. We prefer not to add another picture as the linear transducer is a standard in hip ultrasound and the manuscript already contains quite a lot of display items.

Line126: add for DDH classification

**Response:** We have added the classification in Table 1 (**line: 511, Table 1**)

Line 127: again, if you had explained a bit about the Graf system, then the odd vs even days" would be clearer to the reader

**Response:** We have added the Graf's and the "ABCD" systems explanation. (**lines: 66-73 for Graf, 142-151 for "ABCD", and Table 1**)

Line129: delete "up"

**Response:** It was deleted. (**line: 183**)

Line132: You mean the "examinees" (those who are being examined, not those who are examining). If in doubt, you can also write "to the nurses and physicians"

**Response:** It was corrected (**line:186**)

Line135: interpreted (past tense)

**Response:** Thank you, it was corrected (**line: 189**)

Line137: to make it even more clear you could put "consensual agreement"

**Response:** Thank you, it was corrected (**line:190-191**)

Line 142: Is it "only" or "at least" (meaning that there could be more that one)

**Response:** It was corrected (**line: 196**)

Line146: is it 'only' or "at least" (see above)

**Response:** It was corrected (**line: 200**)

Line 155: closing quotation"

**Response:** It was corrected (**line: 202**)

Line156: opening quotation

**Response:** It was corrected (**line:203**)

Line162: (comma)

**Response:** It was corrected (**line:218**)

Line194: of the transducer? Better to explain it fully.

**Response:** It was clarified (**line: 206-208**)

Line 200: Standard plain cut (need explaining?)

**Response:** It was explained (**line: 68-71**)

Line 219: comma

**Response:** Thank you, it was corrected (**line: 278**)

Line 230: add ‘and’

**Response:** Thank you, it was added (**line: 290**)

Line235: It is OK here but could have been added at the beginning, so that the reader would know more when you cited A....D

**Response:** It was added (**line: 142-151**, **Table1**)

Line 239: Use same [ ] method for citing

**Response:** It was corrected (**line: 299**)

Line245: because

**Response:** It was added (**line: 305**)

Line252: . (period)

**Response:** It was added(**line: 312**)

Line 258 But important to highlight some potential differences, which can help the particular training of nurses (e.g.,, anatomy)

**Response:** It was highlighted (**line: 317-318, 378-379**)

Line284: Use same [ ] method for citing.

**Response:** It was corrected (**line: 344**)

Line306: computerized

**Response:** It was corrected (**line: 364**)

Line 316: do you want to a something like “acceptable” given the limitations you described above?

**Response:** It was modified (**line: 373-379**)

Line 429 Table 2: There could be a minor comment about these changes, which are non-significant probably because of the sample size. It provides an insight into the need for more "anatomical" training for the nurses! (table 2)

**Response:** It was highlighted (**line: 377-378**)

---

**Reviewer 2**

The study designed is to provide an alternative to trained doctors when are not available in detecting DDH in newborns. It needs more clarification and efficient interpretation. The data depicted is not convincing enough. Overall, the aim of the authors to give untrained nurses to diagnose newly Borns with DDH seems a bit risky and not a reliable approach.

**Response:** We agree that it is risky if nurses diagnose newborns with DDH. In our case, nurses do not intend to diagnose DDH (it is usually not allowed). Our study focused on evaluating whether nurses and junior physicians can obtain the correct standard plane of the hip sonograms and identify Graf's "non-Type 1" ("non-Group A") hips.

In Mongolia, the baseline assessment of the hip should be done within two days after birth, before families leave the hospital. This is the standard according to Mongolian guidelines. Under these conditions prevailing in an emerging country with difficult transport routes, screening concepts close to home are indispensable. However, a huge turnover within physicians' teams and a loss of trained doctors due to high internal and international migrations is limiting access in rural areas. Therefore, it might be wise to train nurses since nurses are a more stable group in terms of mobility or changing workplace in the country. Furthermore, it has been shown that nurses can perform ultrasound scans in other settings, specifically in the placement of central [26] and peripheral intravenous lines [26, 28] in patients with difficult access, for the focused assessment in emergency situations [30, 32, 33] and patients with urologic [29, 34], obstetric [31, 35], and cardiac diseases [27]. In order to reduce risks and increase the reliability, a quality control is essential. In Mongolia, a web-based, password-protected platform is used for quality control purposes. The tool enables screeners in all hospitals to upload digital images and annotations exported from the ultrasound machines. Four images (two per hip side; one of them with measured alpha and beta angles according to Graf), a unique identification number, age, sex and diagnosis are required for all examinations. After the upload, quality is controlled by a local expert (a doctor with profound knowledge of the Graf method and at least three years of experience of hip ultrasound and treatment at a provincial hospital). This allowes continuous and reliable reviews of all examinations. In addition, Mongolian supervisors (doctors with profound knowledge of the Graf method and more than 10 years of experience of hip ultrasound and treatment in UB) check examinations from all hospitals and promptly send comments to the experts and screeners.

We added this information to the manuscript. (**line: 99-115, 87-98, 170-171, 209-212**)

The title should be modified to more clear aim and should specify the 'newborns'.

**Response:** The title was modified (**line: 1-3**)

The introduction lacks the critical relevance of DDH, its diagnosis, factors, epidemiological reasons and basic information

**Response:** Thank you for this suggestion. We added all of your requested information to the introduction, table 1 and figure 1 (**lines: 50-56, 66-73, 87-98, 142-151, 206-287, Table 1, Figure 1)**

The information regarding 'ABCD' system should be mentioned in material sand methods and well defined to the new readers.

**Response:** The information regarding 'ABCD' system was added. (**lines: 142-151**)

---

**Reviewer 3**

I would like to express my appreciation for the thoroughness and quality of your work. Your research is both significant and compelling. I was impressed by the clarity of your methodology, the validity of your findings, and the overall organization of your paper.
With my acknowledgement to the strength of your results, I think that your research should include relevant figures, charts, or graphs to visually present your findings and enhance the overall presentation of the results section.

**Response:** Thank you. We added figures. (**Figure 1, 2**)

---

**Reviewer 4**

Overall promising paper. Work can be strengthened by clarification in technique/methods (more in depth description of the doctors/nurses training, handling of hips vs patients), better presentation organization, and better statistical consideration. In particular, the numbers shown suggest there may be some decrease in quality of the exam in nurses compared to doctors that might reach significance if n is increased (see below in conclusion). Power analysis is definitely needed any time a "no difference" in result is proposed.

**Response:** Power calculations indicate that we need to include at least 92 subjects in each group based on the assumptions that 1) the expected proportion of insufficient quality of hip ultrasound of the junior physician group is 10%, 2) the expected proportion of insufficient quality of hip ultrasound of the nurse group is 25%, 3) the alpha level is 0.05, and 4) the required power is 80%. (**line: 227-231**)

Title:
-Might want to include what is being screened for (developmental hip dysplasia).

**Response:** The title was modified accordingly (**line: 1-3**)

Article information:
-The funding information is incomplete. Need which author received the fund, and/or grant number.

**Response:** The first author received a doctoral training support from the Swiss Mongolian Pediatric Project (SMOPP) https://www.sipp.swiss/and the Swiss Association of Pediatric Ultrasound (SVUPP). And we updated this information in the PLOS ONE submission system.

Abstract:
-Base on the data, would not say "can obtain… sonograms… with equal quality." See below in conclusion.

**Response:** it was modified**.** (**line: 41-47**)

Introduction:
-Line 75: "drainage" of knowledge?

**Response:** It was modified (**line: 104-105**)

Methods:
-What is the background of training of the nurses (what kind of schooling)? And of the junior physicians? What kind of physicians are they? Years of experience?

**Response:** The information was added (**line: 124-133**)

-The nurses and junior doctors completed 1 week of introductory courses and 1 week of supervised ultrasound screening. How many hours of courses were there? How many patients (approximately) were screened while supervised? You said 10-20 were "intended," but how many were actually done?

**Response:** It was clarified (line: **156 and 165-168**)

-Was power analysis done?

**Response:** See above (Line: **227-231**)

-Were the right and hips treated as separate subjects? 201 newborns were screened… were the data treated as 201 subjects or 402 hips in the calculations?

**Response:** We used the child and not the hip sonograms (2 sonograms per hip or 4 hip sonograms per child) as the unit of analysis. If a child had hip sonograms with different qualities, we evaluated the child based on the worst quality. (**line: 225-226**)

-Would remove the multivariate modeling. The information in Table 4 (univariate analysis) is sufficiently clear to show the patients prone to have errors and likely source of error. The multivariate analysis does not add much, and doesn't make sense to combined demographic and exam characteristic factors.

**Response:** We removed the multivariate modeling from the paper.

Results:
-The division of Table 2 and Table 3 do not make sense. Table 2 (quality of images) included diagnosis (not a quality metric) and anatomical identification (interpretative metric), but not the factors in Table 3 (tilting and missing lower limb- factors of image quality).

**Response:** We removed Table 2 and Table 3 and combined them as a chart. (**Figure 2**)

Conclusion:
-The numbers presented 96% correct anatomical identification for doctors vs 90% for nurses, 97% correct standard plane vs 91% for nurses nearly reach statistical significance (p = 0.077-0.10). Without power analyses, the conclusion that they are equal in quality is not justified. Would consider possibly rephrasing to say that while there might be a trend of slightly more incorrect technical factors in the nurses group, the overall diagnosis accuracy is similar.

**Response:** Thank you, we modified the conclusion. (**line: 373-379**)

---

**Reviewer 5**

This is an interesting topic. However, it's hard to read and fully understand. Based on my best guess, I have study design and analysis questions.

1. Line 81 states basically trained nurses and trained junior physicians. Are they all graduated from certified medical schools? Can they represent the current nurse or junior physician population in the country?

**Response:** Yes, they all graduated from certified schools. We added this information. (**line: 127-133**) The generalizability of this study into the whole nurses' community of different levels of care is limited because only six nurses and six physicians performed the examinations. We mention the limitations. (**lines: 356-362**)

2. Line 105 states the standard week courses. Could you specify how many hours in total? During the course, were a pretraining test and a post-training test performed? If did, how about performance?

**Response:** The nurses and junior physicians completed 15 hours including practical works (a week) course. There was no pre-training test since all participants had no previous knowledge or experience in Graf's method of hip ultrasound examinations. However, a post-training test was performed and included questions on theoretical and practical

(interpretation and handling) knowledge. The average score of the test was not significantly different between the two groups.
This information was added. (**lines: 155, 157-161**)

3. Line 110 to 113 states around 10 to 20 sonograms. Is there any correlation between the training sonogram number and the final performance of each nurse and physician in the study?

**Response:** We apologize for this mistake. Each nurse and junior physician undertook 10 examinations totaling 20 hips (80 sonograms). It was corrected in the paper. (**line: 165-168**)

4. Table 1. Why did you combine provincial and subprovincial groups? Since your paper tried to convince us that standard training could help rural regions. The subprovincial group performance is more important and better to make a point. Could you explain why you combined two groups?

**Response:** The provincial and subprovincial groups are combined in **Table 1** because we wanted to keep the table on characteristics brief and did not use the geographic location in the analysis.

5. Table 2. There are two numbers 0.245 and 0.100, on the line of Anatomical identification. Could you please explain it?

**Response:** We removed this table from the paper following the suggestions of other reviewers.

Data Review URL

Click here to download Data Review URL
https://www.dropbox.com/scl/fi/ds8ykbc2j6z3e4ygalunn/Data-Review-URL.zip?rlkey=0wa7jegu59592r53h97mmdbvk&dl=0
