# Supplementary material for: Comparison of quality and interpretation of newborn ultrasound screening examinations for developmental dysplasia of the hip by basically trained nurses and junior physicians with no previous ultrasound experience
Source: PLoS One. 2024 Apr 18;19(4):e0300753. doi: 10.1371/journal.pone.0300753 (PMC11025947; doi:10.1371/journal.pone.0300753)
Supplement: Attachment — Submitted filename: comments.docx [file pone.0300753.s003.docx]

PLOS ONE peer review for Comparison of quality and interpretation of hip ultrasound screening examinations by basically trained nurses to junior physicians with no previous ultrasound experience (PONE-D-23-02980)

This is an interesting topic. However, it’s hard to read and fully understand. Based on my best guess, I have study design and analysis questions.

1. Line 81 states basically trained nurses and trained junior physicians. Are they all graduated from certified medical schools? Can they represent the current nurse or junior physician population?
2. Line 105 states the standard week courses. How many hours total? During the course, were a pretraining test and a post-training test performed? If did, how about performance?
3. Line 110 to 113 states around 10 to 20 sonograms. Is there any correlation between the training sonogram number and the final performance of each nurse and physician in the study?
4. Table 1. Why did you combine provincial and subprovincial groups? Since your paper tried to convince us that standard training could help rural regions. The subprovincial group performance is more important and better to make a point. To better pursue your goal, the comparison among three groups (referral, provincial, and subprovincial) would provide a strong argument.
5. Table 2. There are two numbers 0.245 and 0.100, on the line of Anatomical identification. Could you please explain it?
