# Supplementary material for: Comparison of quality and interpretation of newborn ultrasound screening examinations for developmental dysplasia of the hip by basically trained nurses and junior physicians with no previous ultrasound experience
Source: PLoS One. 2024 Apr 18;19(4):e0300753. doi: 10.1371/journal.pone.0300753 (PMC11025947; doi:10.1371/journal.pone.0300753)
Supplement: Attachment — Submitted filename: Response to reviewers.docx [file pone.0300753.s004.docx]

**Editor Comments**

The manuscript has been evaluated by five reviewers, and their comments are available below. The reviewers have raised a number of concerns throughout your manuscript that need attention. They recommend additional background information in the Introduction to better introduce the topic. They also feel the Methods section may benefit from further clarification, particularly as it pertains to the education of the trainees, and the inclusion of statistical power calculations which may validate conclusions drawn from your study. It is also suggested to include additional images which may enhance presentation for the reader. Could you please revise the manuscript to carefully address the concerns raised?

**Response:** Thank you and the reviewers very much for the opportunity to submit a revision of our manuscript. We added additional background information, information on the education of the trainees and statistical power calculations, as well as images. Below, we respond to each point raised by the reviewers. If we can provide any additional information or make any other changes, please do not hesitate to let us know.

The manuscript has important value for dissemination, given the needs at the primary care level in many countries. The study and results are well designed and explained. However, perhaps from the language and writing parts there are several unclear expressions that may confuse the readers. Thus, I have made several suggestions so that you and authors can review, revise, correct and then re-submit. Thank you.

**Response:** Thank you for your support. We are not sure which suggestions came from you as the comments of you and the reviewers were submitted in three parts (text in the e-mail, PDF and Word file). We respond to each point from all three parts below and carefully address the concerns raised.

**Reviewer 1**

There are several issues in this manuscript. Please see the attached PDF sent with the review, where I place several suggestions to improve the data analysis and manuscript writing, for better understanding and integrity.

**Response:** Thank you for your comments. They were very helpful in crafting our revision.

Line 2: It seems better to change the word to “and” instead of “to” (to avoid confusion in readers)

**Response:** We have modified the title of the manuscript **(lines: 1-3)**

Line48: It would be good to start with some form of "prevalence" of dysplasia and other developmental problems in the country and region, to put into context what you will be developing...

**Response:** The information was added (**lines: 50-56**)

Line48: Add something like "...in the neonates" to make it clear to whom it is done.

**Response:**  It was added (**line: 57**)

Line 52: "the Graf method" (it would be good to add one sentence briefly explaining what it is, for the benefit of readers who are not very familiar with it).

**Response:**  Information and picture were added (**lines: 66-73 and Figure 1**)

Line 65: insert “the ”

**Response:** It was corrected (**line: 82**)

Line 66: The Swiss- Mongolian Pediatric Project

**Response:** Thank you. It was corrected (**line: 83**)

Line 67: a humanitarian

**Response:** It was corrected (**line: 83**)

Line 67: Add braces to make it more clear

**Response:** It was changed. (**line: 84**)

Line 68: Add “as a way to help correct the skeletal system” (to make the phrase clearer)

**Response:** The phrase was added (**lines: 85-86**)

Line 79: add “program”

**Response:**  It was added (**line: 109**)

Line80: This is not clear. Did you mean to say "This study will be followed..." (because until now, there is no mention of a "study" so this surprises the reader). Please correct the phrase...

**Response:** The sentences were modified (**lines: 109-115**)

Line82: “and by”

**Response:**  It was corrected (**line: 114**)

Line 88: from March to December 2021”,

**Response:** It was corrected (**line: 120**)

Line89: "the Mongolia Ministry of Health (MOH)"

**Response:** It was corrected (**lines:120-121**)

Line 92: "self-selected" means they volunteered? Also, were there any criteria for such selection (e.g., more experienced, working at least one year, etc.?). It is important to give the reader as much information about this selection as possible.

**Response:** We agree that the term “self-selected” was unclear. We adapted the paragraph (**lines:** **124-127**)

Line 100: Again, explain a little bit about this ABCD system” for the benefit readers…

**Response:** The explanation was added (**lines: 142-151, Table 1**)

Line 101: "The selected nurses and junior physicians, who had no prior knowledge or experience in hip sonography" (to make it clearer and accurate).

**Response:** We removed the sentence and now mention the inclusion criterion under “Study participants”. (**lines: 124-127**)

Line110: This is NOT clear. "It was intended" means it was DONE or was put as an objective? Does it mean that they at least undertook 10 sonograms to gain experience? This has to be clear, otherwise the reader doesn't know what happened...

**Response:** Thank you, it was clarified (**lines:165-168**)

Line116: "met" (past tense)

**Response:**  It was corrected (**line: 171**)

Line117: "were" (past tense)

**Response:**  It was corrected (**line: 172**)

Line123: add “ultrasound machine”

**Response:** It was added (**lines:177-178**)

Line 124: It is not absolutely necessary, but you could add a picture/graph to make it clearer - ?

**Response:**  Thank you for the suggestion. We prefer not to add another picture as the linear transducer is a standard in hip ultrasound and the manuscript already contains quite a lot of display items.

Line126: add for DDH classification

**Response:**  We have added the classification in Table 1 (**line: 511, Table 1**)

Line 127: again, if you had explained a bit about the Graf system, then the odd vs even days” would be clearer to the reader

**Response:** We have added the Graf’s and the “ABCD” systems explanation. (**lines: 66-73 for Graf, 142-151 for “ABCD”, and Table 1**)

Line129: delete “up”

**Response:** It was deleted. (**line: 183**)

Line132: You mean the "examinees" (those who are being examined, not those who are examining). If in doubt, you can also write "to the nurses and physicians"

**Response:** It was corrected (**line:186**)

Line135: interpreted (past tense)

**Response:** Thank you, it was corrected (**line: 189**)

Line137: to make it even more clear you could put “consensual agreement”

**Response:** Thank you, it was corrected (**line:190-191**)

Line 142: Is it “only” or “at least” (meaning that there could be more that one)

**Response:** It was corrected (**line: 196**)

Line146: is it ‘only’ or “at least” (see above)

**Response:** It was corrected (**line: 200**)

Line 155: closing quotation”

**Response:** It was corrected (**line: 202**)

Line156: opening quotation

**Response:** It was corrected (**line:203**)

Line162: (comma)

**Response:** It was corrected (**line:218**)

Line194: of the transducer? Better to explain it fully.

**Response:** It was clarified (**line: 206-208**)

Line 200: Standard plain cut (need explaining?)

**Response:**  It was explained (**line: 68-71**)

Line 219: comma

**Response:** Thank you, it was corrected (**line: 278**)

Line 230: add ‘and’

**Response:** Thank you, it was added (**line: 290**)

Line235: It is OK here but could have been added at the beginning, so that the reader would know more when you cited A....D

**Response:** It was added (**line: 142-151**, **Table1**)

Line 239: Use same [ ] method for citing

**Response:** It was corrected (**line: 299**)

Line245: because

**Response:** It was added (**line: 305**)

Line252: . (period)

**Response:** It was added(**line:** **312**)

Line 258 But important to highlight some potential differences, which can help the particular training of nurses (e.g.,, anatomy)

**Response:** It was highlighted (**line: 317-318, 378-379**)

Line284: Use same [ ] method for citing.

**Response:** It was corrected (**line: 344**)

Line306: computerized

**Response:** It was corrected (**line: 364**)

Line 316: do you want to a something like “acceptable” given the limitations you described above?

**Response:** It was modified (**line: 373-379**)

Line 429 Table 2: There could be a minor comment about these changes, which are non-significant probably because of the sample size. It provides an insight into the need for more "anatomical" training for the nurses! (table 2)

**Response:** It was highlighted (**line: 377-378**)

**Reviewer 2**

The study designed is to provide an alternative to trained doctors when are not available in detecting DDH in newborns. It needs more clarification and efficient interpretation. The data depicted is not convincing enough. Overall, the aim of the authors to give untrained nurses to diagnose newly Borns with DDH seems a bit risky and not a reliable approach.

**Response:** We agree that it is risky if nurses diagnose newborns with DDH. In our case, nurses do not intend to diagnose DDH (it is usually not allowed). Our study focused on evaluating whether nurses and junior physicians can obtain the correct standard plane of the hip sonograms and identify Graf’s “non-Type 1” (“non-Group A”) hips.

In Mongolia, the baseline assessment of the hip should be done within two days after birth, before families leave the hospital. This is the standard according to Mongolian guidelines. Under these conditions prevailing in an emerging country with difficult transport routes, screening concepts close to home are indispensable. However, a huge turnover within physicians’ teams and a loss of trained doctors due to high internal and international migrations is limiting access in rural areas. Therefore, it might be wise to train nurses since nurses are a more stable group in terms of mobility or changing workplace in the country. Furthermore, it has been shown that nurses can perform ultrasound scans in other settings, specifically in the placement of central [26] and peripheral intravenous lines [26, 28] in patients with difficult access, for the focused assessment in emergency situations [30, 32, 33] and patients with urologic [29, 34], obstetric [31, 35], and cardiac diseases [27]. In order to reduce risks and increase the reliability, a quality control is essential. In Mongolia, a web-based, password-protected platform is used for quality control purposes. The tool enables screeners in all hospitals to upload digital images and annotations exported from the ultrasound machines. Four images (two per hip side; one of them with measured alpha and beta angles according to Graf), a unique identification number, age, sex and diagnosis are required for all examinations. After the upload, quality is controlled by a local expert (a doctor with profound knowledge of the Graf method and at least three years of experience of hip ultrasound and treatment at a provincial hospital). This allowes continuous and reliable reviews of all examinations. In addition, Mongolian supervisors (doctors with profound knowledge of the Graf method and more than 10 years of experience of hip ultrasound and treatment in UB) check examinations from all hospitals and promptly send comments to the experts and screeners.

We added this information to the manuscript. (**line: 99-115, 87-98, 170-171, 209-212**)

The title should be modified to more clear aim and should specify the 'newborns'.

# **Response:** The title was modified (**line: 1-3**)

The introduction lacks the critical relevance of DDH, its diagnosis, factors, epidemiological reasons and basic information

**Response:** Thank you for this suggestion. We added all of your requested information to the introduction, table 1 and figure 1 (**lines: 50-56, 66-73, 87-98, 142-151, 206-287,** **Table 1, Figure 1)**

The information regarding 'ABCD' system should be mentioned in material sand methods and well defined to the new readers.

**Response:**  The information regarding 'ABCD' system was added. (**lines: 142-151**)

**Reviewer 3**

I would like to express my appreciation for the thoroughness and quality of your work. Your research is both significant and compelling. I was impressed by the clarity of your methodology, the validity of your findings, and the overall organization of your paper.
With my acknowledgement to the strength of your results, I think that your research should include relevant figures, charts, or graphs to visually present your findings and enhance the overall presentation of the results section.

**Response:** Thank you. We added figures. (**Figure 1, 2**)

**Reviewer 4**

Overall promising paper. Work can be strengthened by clarification in technique/methods (more in depth description of the doctors/nurses training, handling of hips vs patients), better presentation organization, and better statistical consideration. In particular, the numbers shown suggest there may be some decrease in quality of the exam in nurses compared to doctors that might reach significance if n is increased (see below in conclusion). Power analysis is definitely needed any time a "no difference" in result is proposed.

**Response:** Power calculations indicate that we need to include at least 92 subjects in each group based on the assumptions that 1) the expected proportion of insufficient quality of hip ultrasound of the junior physician group is 10%, 2) the expected proportion of insufficient quality of hip ultrasound of the nurse group is 25%, 3) the alpha level is 0.05, and 4) the required power is 80%. (**line: 227-231**)

Title:
-Might want to include what is being screened for (developmental hip dysplasia).

**Response:**  The title was modified accordingly (**line: 1-3**)

Article information:
-The funding information is incomplete. Need which author received the fund, and/or grant number.

**Response:** The first author received a doctoral training support from the Swiss Mongolian Pediatric Project (SMOPP) https://www.sipp.swiss/and the Swiss Association of Pediatric Ultrasound (SVUPP). And we updated this information in the PLOS ONE submission system.

Abstract:
-Base on the data, would not say “can obtain… sonograms… with equal quality.” See below in conclusion.

**Response:** it was modified**.** (**line: 41-47**)

Introduction:
-Line 75: “drainage” of knowledge?

**Response:** It was modified (**line: 104-105**)

Methods:
-What is the background of training of the nurses (what kind of schooling)? And of the junior physicians? What kind of physicians are they? Years of experience?

**Response:**  The information was added (**line: 124-133**)

-The nurses and junior doctors completed 1 week of introductory courses and 1 week of supervised ultrasound screening. How many hours of courses were there? How many patients (approximately) were screened while supervised? You said 10-20 were “intended,” but how many were actually done?

**Response:** It was clarified (line: **156 and 165-168**)

-Was power analysis done?

**Response:** See above (Line: **227-231**)

-Were the right and hips treated as separate subjects? 201 newborns were screened… were the data treated as 201 subjects or 402 hips in the calculations?

**Response:**  We used the child and not the hip sonograms (2 sonograms per hip or 4 hip sonograms per child) as the unit of analysis. If a child had hip sonograms with different qualities, we evaluated the child based on the worst quality. (**line: 225-226**)

-Would remove the multivariate modeling. The information in Table 4 (univariate analysis) is sufficiently clear to show the patients prone to have errors and likely source of error. The multivariate analysis does not add much, and doesn’t make sense to combined demographic and exam characteristic factors.

**Response:** We removed the multivariate modeling from the paper.

Results:
-The division of Table 2 and Table 3 do not make sense. Table 2 (quality of images) included diagnosis (not a quality metric) and anatomical identification (interpretative metric), but not the factors in Table 3 (tilting and missing lower limb- factors of image quality).

**Response:** We removed Table 2 and Table 3 and combined them as a chart. (**Figure 2**)

Conclusion:
-The numbers presented 96% correct anatomical identification for doctors vs 90% for nurses, 97% correct standard plane vs 91% for nurses nearly reach statistical significance (p = 0.077-0.10). Without power analyses, the conclusion that they are equal in quality is not justified. Would consider possibly rephrasing to say that while there might be a trend of slightly more incorrect technical factors in the nurses group, the overall diagnosis accuracy is similar.

**Response:** Thank you, we modified the conclusion. (**line: 373-379**)

**Reviewer 5**

This is an interesting topic. However, it’s hard to read and fully understand. Based on my best guess, I have study design and analysis questions.

1. Line 81 states basically trained nurses and trained junior physicians. Are they all graduated from certified medical schools? Can they represent the current nurse or junior physician population in the country?

**Response:** Yes, they all graduated from certified schools. We added this information. (**line: 127-133**) The generalizability of this study into the whole nurses’ community of different levels of care is limited because only six nurses and six physicians performed the examinations. We mention the limitations. (**lines: 356-362**)

2. Line 105 states the standard week courses. Could you specify how many hours in total? During the course, were a pretraining test and a post-training test performed? If did, how about performance?

**Response:** The nurses and junior physicians completed 15 hours including practical works (a week) course. There was no pre-training test since all participants had no previous knowledge or experience in Graf’s method of hip ultrasound examinations. However, a post-training test was performed and included questions on theoretical and practical (interpretation and handling) knowledge. The average score of the test was not significantly different between the two groups.

This information was added. (**lines: 155, 157-161**)

3. Line 110 to 113 states around 10 to 20 sonograms. Is there any correlation between the training sonogram number and the final performance of each nurse and physician in the study?

**Response:** We apologize for this mistake. Each nurse and junior physician undertook 10 examinations totaling 20 hips (80 sonograms). It was corrected in the paper. (**line: 165-168**)

4. Table 1. Why did you combine provincial and subprovincial groups? Since your paper tried to convince us that standard training could help rural regions. The subprovincial group performance is more important and better to make a point. Could you explain why you combined two groups?

**Response:** The provincial and subprovincial groups are combined in **Table 1** because we wanted to keep the table on characteristics brief and did not use the geographic location in the analysis.

5. Table 2. There are two numbers 0.245 and 0.100, on the line of Anatomical identification. Could you please explain it?

**Response:** We removed this table from the paper following the suggestions of other reviewers.
