# Supplementary material for: Comparison of quality and interpretation of newborn ultrasound screening examinations for developmental dysplasia of the hip by basically trained nurses and junior physicians with no previous ultrasound experience
Source: PLoS One. 2024 Apr 18;19(4):e0300753. doi: 10.1371/journal.pone.0300753 (PMC11025947; doi:10.1371/journal.pone.0300753)
Supplement: Attachment — Submitted filename: comment_1.docx [file pone.0300753.s005.docx]

Line 60 stated that the Graf method is examiner-independent. However, line 37 said the most common errors among the groups were a missing lower limb, wrong measurement lines, and technical problems. Ultrasound, as known, is heavily dependent on examiners.
