# Supplementary material for: Comparison of quality and interpretation of newborn ultrasound screening examinations for developmental dysplasia of the hip by basically trained nurses and junior physicians with no previous ultrasound experience
Source: PLoS One. 2024 Apr 18;19(4):e0300753. doi: 10.1371/journal.pone.0300753 (PMC11025947; doi:10.1371/journal.pone.0300753)
Supplement: Attachment — Submitted filename: Nurse_PONE_ point by point answers_2_SE.docx [file pone.0300753.s007.docx]

**Journal Requirements**

Please review your reference list to ensure that it is complete and correct. If you have cited papers that have been retracted, please include the rationale for doing so in the manuscript text, or remove these references and replace them with relevant current references. Any changes to the reference list should be mentioned in the rebuttal letter that accompanies your revised manuscript. If you need to cite a retracted article, indicate the article’s retracted status in the References list and also include a citation and full reference for the retraction notice.

**Response:** The reference list was re-checked and corrected: two mistakenly retracted papers were removed and replaced them with relevant current references **(Ref 15- line: 413-417 and Ref 24: lines: 446-447)**, one paper was added due to additional explanations in the text (**Ref 20**- **lines: 431-434**).

**Additional Editor Comments**

Thank you for revising your manuscript according to the reviewers' concerns. The reviewers have a few additional minor requests to improve the quality of the language and clarity of the explanations outline below and attached. I note that you modified your title in the manuscript file - please ensure you have also updated the title in the online submission form.

**Response:** Thank you very much for the opportunity to submit a revision of our manuscript. We now updated the title in the online submission form. We will respond to the points raised by the reviewers below.

**Reviewer 1**

Line-119: This concept is unclear. What "other criteria", and why were they considered "obsolete" (irrelevant? outdated?)? Explain or delete if not important.

**Response:** Thank you for your comments. We deleted it. **(line: 118)**

Line-131: through a...

**Response:** We have added. **(line: 128)**

Line-137: From this sentence forward, it is not clear why there are negative concepts put here (eg., "subtypes...irrelevant", "not practical... to learn... subtypes and... treatments... because... hips are treated in the same way". What are you trying to say? When you put that the Graf system "required some adjustments...", is that because it needed to be clearer for the purposes of the study only, or more generally...? Please double-check the paragraph to make is clear and sensible...

**Response:** We agree that the phrasing of the paragraph was not sensible. We also clarified that the ABCD system is a national standard. We adjusted the manuscript: “The “ABCD” system is a diagnostic and therapeutic framework based on Graf’s technique. SMOPP established the system as a nationally recognized standard, ensuring it is user-friendly for screeners in maternity hospitals and capable of distinguishing between groups essential for both controls and treatment. The system is entirely based on the method described by Graf and translates his initial differentiation into four groups: Group A includes Graf Type 1; Group B includes Graf Type 2a; Group C includes Graf Types 2c, D and 3; and Group D includes Graf Type 4 (Table 1).” (**lines: 135-140**)

Line-148: ADD SOME EXPLANATION BECAUSE IT WILL BECOME AN IMPORTANT ERROR: (i.e., probe incorrectly positioned, which produces distortions and misleading images),

**Response:**  We added the explanation: “…tilting errors (inaccuracies due to improper transducer positioning resulting in distortions and misleading images), …” (**lines: 143-144**)

Line-154: You should not put this here, because these are "Results" already!

**Response:**  Thank you, we moved the sentence to the results section (**line: 150 and lines: 226-227**)

Line-160: each

**Response:** Corrected (**line: 154-155**)

Line-160: , (comma)

**Response:**  Corrected (**line: 155**)

Line-168- Strikethrough

**Response:** It was changed (**lines: 161-163**)

Line-177-What is this? It's not clear...do you mean something like "the procedures and management done by both groups, depending on the results of the ultrasounds, followed the national guideline..."?

**Response:** We corrected the phrase: “ …Monthly ultrasound follow-up control until full maturation and management done by both groups, depending on the results of the ultrasounds, followed the national guideline.” (**lines: 171-173**)

Line-184-posterior

**Response:** It was added (**line: 179**)

Line-196- Which are these criteria? Can they (or a summary of them) be put here?

Response: We added the following explanation: “baseline - a vertical line, parallel to the ossified lateral wall of the ilium; bony roof line - a line drawn from the inferior edge of the osseous acetabulum, the inferior iliac margin, at the triradiate cartilage roof to the most lateral point on the ilium, the superior osseous rim; and cartilaginous roof line - a line traced along the cartilaginous acetabulum's roof, extending from the acetabulum's lateral osseous edge to the center of the labrum” **(lines: 191-196, Reference lines 429-432)**

Line-201-This is important to describe here.

**Response:** The sentences were modified. (**lines: 201-202**)

Line-203-Not clear. Do you mean something like: "based on the results of the tomography the nurses and junior physicians were requested to arrive at a final diagnosis, utilizing one of two terms: "Group A" (Graf's Type 1 hip) or "Non-Group A" (Graf's non-Type 1 hip)"?

**Response:**  Thank you for the correction. We corrected the text accordingly. (**lines: 203-205**)

Line-211- probability

**Response:** It was added (**line: 213**)

Line-244- of the probe, which deflects the ultrasound beam and can produce a misleading and incorrect image

**Response:** Thank you, it was clarified: “A common error among the groups was the tilting of the transducer, which deflects the ultrasound beam and can produce a misleading and incorrect image”. (**lines:246-247**)

Line-245- of

**Response:** It was added (**lines: 248**)

Line-256- high

**Response:** It was added (**line: 259**)

Line-259- of the probe

**Response:** It was added. (**line: 262**)

Line-292- higher numbers,

**Response:** Thank you, it was clarified (**lines:295-296**)

Line-297-high

**Response:**  It was corrected (**line: 300**)

Line-330-the ultrasound probe

**Response:**  It was added (**line: 336**)

Line-357-You need to add a summative conclusion, something like: "despite its limitations, this study seems to confirm the nurses could be authorized to carry out hip ultrasound procedures in order to refer those newborns requiring more specialized treatments to a higher level health establishment, and potentially be able to reduce the occurrence and prevalence of hip dysplasia and disability in developing countries..."

**Response:** Thank you for the comment. We added your suggestion to the text. (**lines:364-368**)

Table-1- Type D- What is this? No "angle"?

**Response:**  D hips are fully dislocated hips. Therefore, the measurements are impossible. We added «Measurement is impossible» to table 1. **(line 506)**

Table-3- Univariate (was misspelled)

**Response:**  Thank you for the correction. It was corrected. (**line: 514, Table 1**)

**Reviewer 3**

Through my reading this manuscript, I found a clear, correct, and unambiguous language. Moreover, in this review, all the previous grammatical errors were also corrected.
Therefore, I accept this paper for publication.

**Response:** Thank you very much.

**Reviewer 4**

**INTRODUCTION**

Line 64: standard PLANE instead of plain? Might want to rephrase as "The method uses a coronal ultrasound image through the center of the acetabulum..."

**Response:** Thank you for the suggestion. We corrected the text accordingly. (**lines: 64-65**)

Line 88, 91 Instead of "profound knowledge," would consider "good knowledge," "experienced in," or "proficient in"

**Response:** It was changed to “good knowledge”. (**line: 88**)

**METHODS**

Line 119 "Other criteria ... " Would remove this sentence. It is confusing and does not add additional information.

**Response:** It was removed. (**line: 118**)

Line 219 Would combine (1) and (2) into "difference in quality of ultrasound of 15%.

**Response:** It was combined. (**line: 220-221**)

**RESULT**

Line 249 "the incorrect diagnosis was significantly more likely to be 'ASSOCIATED WITH' smaller birth weight of newborns..." Correlation and causation are not the same.

**Response:** Thank you, it was corrected. (**line: 252**)

**DISCUSSION**

Would acknowledge that your detection power is limited based on your power analysis (need ~15% difference to be detected).

**Response:** It was acknowledged in the limitation. (**lines: 356-357**)

It might be worth noting that in some places in the world, such as the US, ultrasounds are generally performed by sonographers (technologists) and interpreted by physicians.

**Response:** It was added. (**line: 329-331**)

**TABLE**

Table 3: Incorrect diagnosis total should be 19, not 9

**Response:** Thank you. It was corrected. (**line: 515, Table 3**)

**FIGURE**

Figure 1A and 1B: Image in 1A is too zoomed in, making it difficult to see the position of the femoral head and worsening image quality.

**Response:** The image was replaced. (**line: 500, Figure 1A**)

**Reviewer 5**

Could you please correct the contradictory between line 60 and line 37? Ultrasound is an examiner-dependent modality. That is why examiners should be very trained and certified. Line 60 stated that the Graf method is examiner-independent. However, line 37 said the most common errors among the groups were a missing lower limb, wrong measurement lines, and technical problems. Ultrasound, as known, is heavily dependent on examiners.

**Response:** You are right. We deleted “… examiner independent…” from the text. (**line: 60**)
